# Nanostructured and oriented metal–organic framework films enabling extreme surface wetting properties

**DOI:** 10.3762/bjnano.10.196

**Published:** 2019-10-09

**Authors:** Andre Mähringer, Julian M Rotter, Dana D Medina

**Affiliations:** 1 Department of Chemistry, Ludwig-Maximilians-Universität (LMU), Butenandtstr. 11, 81377 Munich, Germany; 2 Nanosystems Initiative Munich (NIM) and Center for NanoScience (CeNS), Schellingstr. 4, 80799 Munich, Germany

**Keywords:** antifog, antifouling, biomimetic coatings, metal–organic frameworks (MOFs), superhydrophilic, superoleophobic, thin films, vapor-assisted conversion

## Abstract

We report on the synthesis of highly oriented and nanostructured metal–organic framework (MOF) films featuring extreme surface wetting properties. The Ni- and Co- derivatives of the metal–catecholate series (M-CAT-1) were synthesized as highly crystalline bulk materials and thin films. Oriented pillar-like nanostructured M-CAT-1 films exhibiting pronounced needle-like morphology on gold substrates were established by incorporating a crystallization promoter into the film synthesis. These nanostructured M-CAT-1 MOF films feature extreme wetting phenomena, specifically superhydrophilic and underwater superoleophobic properties with water and underwater oil-contact angles of 0° and up to 174°, respectively. The self-cleaning capability of the nanostructured, needle-like M-CAT-1 films was illustrated by measuring time-dependent oil droplet rolling-off a tilted surface. The deposition of the nanostructured Ni-CAT-1 film on a large glass substrate allowed for the realization of an efficient, transparent, antifog coating, enabling a clear view even at extreme temperature gaps up to ≈120 °C. This work illustrates the strong link between MOF film morphology and surface properties based on these framework materials.

## Introduction

Over millions of years, plants and animals have evolved a spectrum of surface designs enabling specific wetting properties tailored for their survival in extreme conditions [1–4]. In plants, the unique surface architecture of the lotus leaf enables superhydrophobic and self-cleaning properties for sustaining efficient photosynthesis, even in polluted environments [5–7]. In the realm of animals, mosquitos utilize an antifog coating covering their eyes for clear vision in high humidity regions [8]. The fogstand beetles employ an antifog coating for enhancing water vapor harvesting abilities in dry climates [9]. In marine life, fish protect themselves from oil-polluted environments or avoid biofouling due to oil-repellent designs and self-cleaning capabilities of their skin [10–12]. These intriguing superhydrophilic or superoleophobic surface characteristics are obtained by the combination of a precise chemical composition and hierarchical microstructuring of the surface [13–16]. Nowadays, modern surface technologies such as antifog or antifouling, oil-repellant coatings, self-cleaning surfaces and water-harvesting systems are inspired by nature’s designs [17–26]. The synthesis of artificial superhydrophilic surfaces can be achieved by a variety of routes, for example, sol–gel synthesis, electrochemical deposition, anodization, electrochemical polymerization, electrospinning, plasma treatment, chemical or hydrothermal methods, vapor deposition, layer-by-layer assembly or laser ablation [19,27–39]. However, the development of a straightforward and versatile bottom-up synthesis scheme enabling tunable surface morphologies for controlled wetting properties is still challenging and highly desired.

Metal–organic frameworks (MOFs) are porous, crystalline materials featuring a great structural and chemical diversity [40–45]. Thereby, they are attractive synthesis targets for a large variety of applications, including gas storage and separation, chemical sensing, thermoelectrics, capacitors, transistors or photovoltaics [46–52]. Due to their exceptional variety of structural properties and functions, MOFs are intriguing candidates for the design and synthesis of coatings combining a superhydrophilic, superhydrophobic, superoleophilic or superoleophobic character with desired features such as light filtering, hosting cavities, electrical conductivity, etc. In the literature, the synthesis procedures of MOF powders with encoded wetting properties have been reported [53–60]. Typically, the desired wetting properties in MOFs were chemically induced by a post-synthesis modification or by including building blocks decorated with hydrocarbon side chains [58,61–64]. Very recently, a copper mesh decorated with hybrid Cu(OH)_2_/MOF-nanobrushes showed underwater contact angles suggesting superoleophobic properties [65]. However, the use of well-defined MOF films for encoding on-surface wetting properties is yet to be revealed.

Here we report the synthesis of highly oriented, nanostructured MOF films that mimic architectures observed in nature, resulting in highly unusual, extreme surface wetting properties. For the synthesis, the Ni- and Co-derivatives of the metal–catecholate series (M-CAT-1) were selected [66]. First, these M-CAT-1 derivatives were synthesized as highly crystalline bulk materials. The M-CAT-1 surface energy was estimated by contact angle measurements using pellet samples exposing nonoriented crystallites on the surface. Next, oriented and compact Co- and Ni-CAT-1 films were deposited on gold surfaces by vapor-assisted conversion (VAC). These films showed enhanced hydrophilic and underwater oleophobic properties compared to the corresponding pressed pellet samples.

Oriented, nanostructured M-CAT-1 films exhibiting a pronounced needle-like morphology on gold substrates were obtained by adding a crystallization promotor into the VAC precursor solution. These nanostructured M-CAT-1 MOF films feature extreme wetting phenomena, specifically superhydrophilic and underwater superoleophobic properties with water and underwater oil-contact angles of 0° and up to 174°, respectively. The self-cleaning capability of the nanostructured, needle-like Ni- and Co-CAT-1 films was illustrated by measuring time-dependent oil droplet rolling-off a tilted surface. The deposition of the nanostructured Ni-CAT-1 film on large glass substrates allowed for the realization of an efficient antifog coating. These antifog coatings enabled clear vision, even at large temperature differences of up to 120 °C.

## Results and Discussion

**Figure 1:**
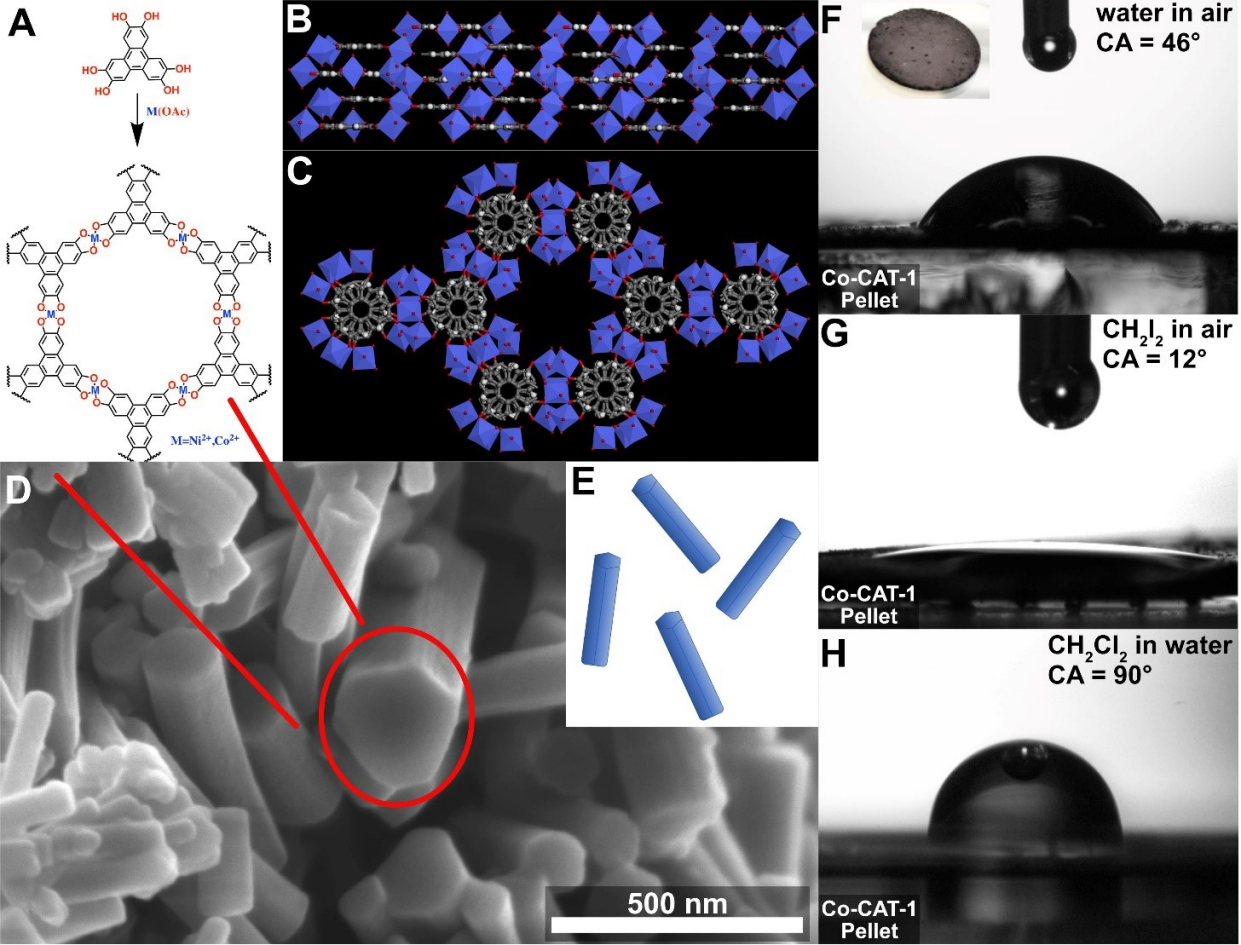
A) Synthesis scheme of M-CAT-1 using 2,3,6,7,10,11-hexahydroxytriphenylene (HHTP) and the respective metal acetate under solvothermal conditions. B) Side view of the Co-CAT-1 crystal structure illustrating infinite layers of HHTP_2_Co_3_. C) View along the crystallographic *c*-axis highlighting the hexagonal pore channels. D) SEM micrograph showing the needle-like crystallite morphology, which is sketched in E) as elongated hexagonally shaped monoliths. F) Water contact angle (WCA) measured at the solid/air interface of a Co-CAT-1 pellet. G) Diiodomethane CA measured on the same pellet. H) Underwater CA measured at the solid/water interface on the identical pelletized sample.

The M-CAT-1 MOFs are crystalline, microporous structures obtained by a solvothermal reaction of metal precursors and the organic building block 2,3,6,7,10,11-hexahydroxytriphenylene (HHTP) as a dark precipitate. The M-CAT-1 crystallites exhibit a hexagonally faceted, needle-like morphology with crystal cross-sections of 30–40 nm and several micrometers in length (Figure 1). In addition, the M-CAT-1 materials are electroactive featuring electrical conductivity values of up to 10^−2^–10^−3^ S cm^−1^ for pressed powder pellets and oriented thin films, respectively [67]. However, the wetting properties of these MOFs both as pristine powders and as films have not been explored.

For our study, we focused on Ni- and the Co-CAT-1, since their structure is well-characterized and elucidated by diffraction methods, serving as a good basis for understanding their growth behavior and morphology. To assess the wetting properties of Ni- and Co-CAT-1, we investigated the interaction of the bulk materials pressed as compact pellets with different polar and apolar liquids. For this purpose, we synthesized M-CAT-1 bulk materials following our recently reported solvothermal synthesis procedure (for more details see Figure S3.1, Supporting Information File 1) [67]. Subsequently, the obtained MOF powders were collected, activated under dynamic vacuum and pressed into crystalline pellet samples (1 cm diameter, thickness 500 µm; 100 mg activated MOF, roughness (RMS): 37 nm). Scanning electron microscope (SEM) micrographs reveal densely packed and randomly oriented MOF crystallites throughout the sample (see Figure S3.2, Supporting Information File 1). For the contact angle (CA) measurements at the solid/air interface we chose diiodomethane as a nonpolar organic liquid, which exhibits a relatively high surface tension, and water as a polar liquid (see Figure 1). CA measurements on M-CAT-1 pellets reveal shallow angles of about 12° for diiodomethane and WCA values of about 46°. This illustrates that in air the M-CAT-1 materials feature an amphiphilic character, namely hydrophilic and superolephilic properties. This amphiphilic wetting property can be attributed to the high surface energy of the MOF materials. To estimate the corresponding MOF surface energy, we applied Fowker’s theory, where the observed CA between a liquid and a solid is related to the sum of a polar and dispersive components of the liquid’s surface tension [68]. Utilizing the measured CAs of diiodomethane exhibiting solely a dispersive component and water, having a dispersive and polar components, enabled the calculation of the overall surface energy for Ni- and Co-CAT-1 (for detailed discussion see Supporting Information File 1, Table S1). We obtained a surface energy of 64.02 and 62.38 mN/m for the Ni- and Co-CAT-1 pellet samples, respectively, as summarized in Table 1. These values are higher than the surface energy reported for oleophilic polymers which exhibit a surface energy in the range of 20–50 mN/m. However, the estimated MOF surface energy and WCAs are in good agreement with systems such as modified carbonaceous layers, showing values of about 60 mN/m and WCAs of 50° [69]. Superoleophilic wetting properties were also detected for chlorinated oils such as dichloromethane on M-CAT-1 pellets (see Figure S3.3, Supporting Information File 1). This further illustrates the amphiphilic nature of the M-CATs, showing hydrophilic as well as superoleophilic behavior towards several different oils. Interestingly, the underwater oil contact angle (OCA) of diiodomethane on these pellets reveals an inverse wetting phenomenon. Under these conditions, the M-CAT-1 materials reject oil and turn oleophobic with an OCA of 90° (Figure 1H).

**Table 1:** Surface energy (σ_S_) and the respective dispersive (σ_S_^D^) and polar (σ_S_^P^) components estimated for Ni- and Co-CAT-1 pellet samples.

M-CAT-1	σ_S_ [mN/m]	σ_S_^D^ [mN/m]	σ_S_^P^ [mN/m]
			
Ni-CAT-1	64.02	49.69	14.06
Co-CAT-1	62.38	50.30	12.08

### Constructing nanostructured M-CAT-1 surfaces

According to Wenzel’s model, film topography has a strong impact on the wetting properties of a surface [70]. Following this line of thought, we aimed at constructing M-CAT-1 thin films featuring distinct surface morphologies, allowing for the desired surface wetting properties to be achieved. However, on-surface control of MOF film morphology is a challenging task due to the required growth conditions for maintaining key features such as homogeneity, crystallinity and crystal orientation on the surface. In our previous reports, we have demonstrated that the use of a modulator in combination with the choice of a substrate play a crucial role in VAC synthesis and the formation of a crystalline MOF film [71]. In the present study, we introduce the use of a crystallization promoter in the VAC process, solely for inducing distinct film morphology while maintaining crystallinity and crystal orientation on a preselected substrate.

Accordingly, we employed VAC methods for producing both compact and nanostructured M-CAT-1 films (see Figure S5.1, Supporting Information File 1). Briefly, oriented thin films of Ni- and Co-CAT-1 were synthesized on gold substrates by depositing a thin layer of a precursor solution onto a gold substrate. Then, the gold substrate was placed on a glass platform above a solvent bath inside a glass autoclave. Subsequently, the reactor was sealed and placed at 85 °C in a preheated oven. After 12 h of reaction time, the reactor was removed from the oven and allowed to cool down to room temperature. The substrates were recovered, washed and dried under a N_2_ stream, revealing a colored film on the gold substrate. These films typically exhibit thickness of about 150 nm, consisting of highly intergrown oriented crystallites (see Figure S5.1, Supporting Information File 1).

**Figure 2:**
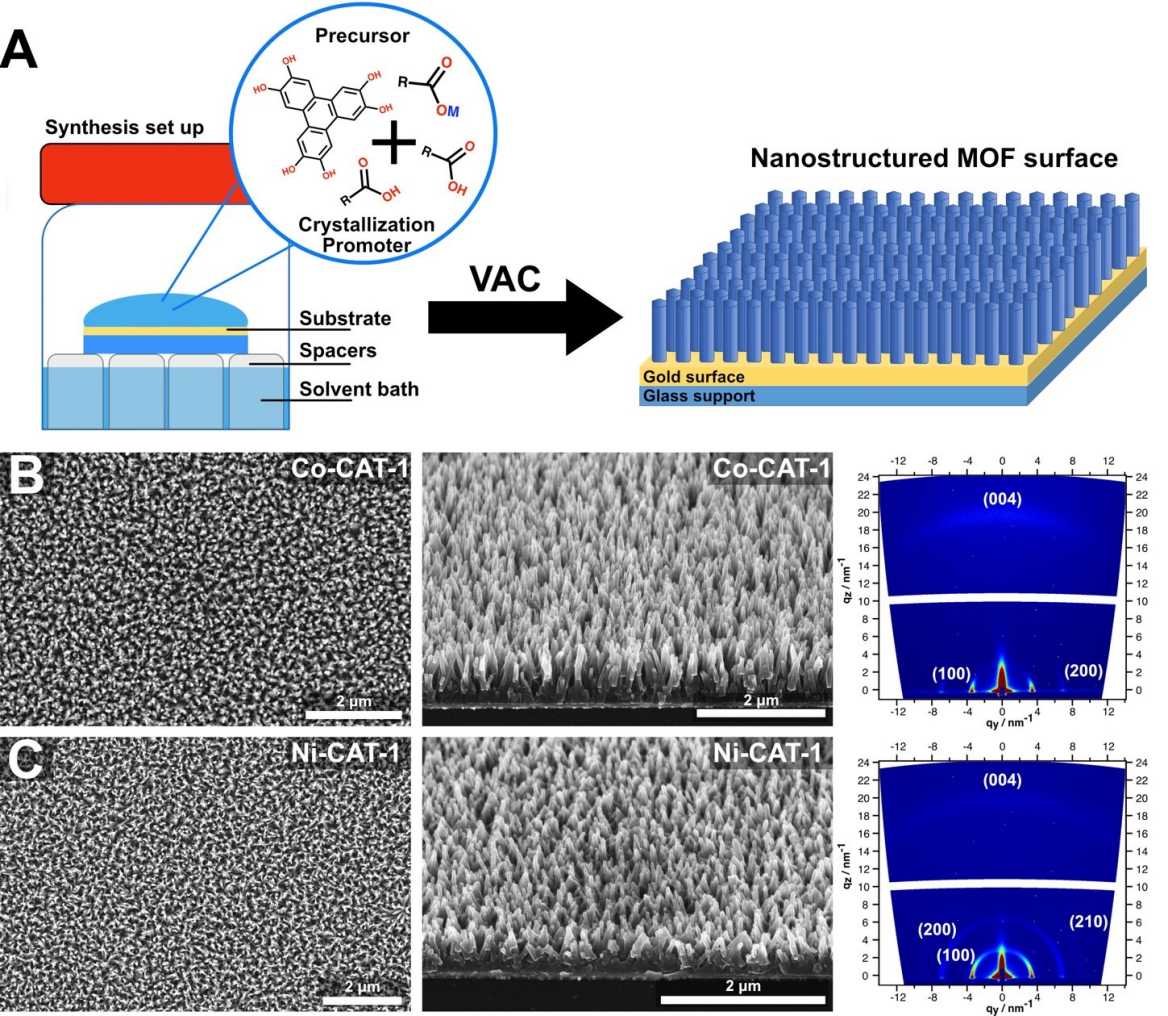
A) A scheme of the vapor-assisted conversion (VAC) set up and the resulting nanostructured films. B) SEM top view, 30° tilted cross-section and the related GIWAXS pattern of the Co-CAT-1 film. C) SEM top view, 30° tilted cross-section momographs and the related GIWAXS pattern of the Ni-CAT-1.

Next, nanostructured pillar-like M-CAT-1 films were synthesized by including carboxylic acids, which served as crystallization promotors in the drop-cast precursor solution layer. Specifically, we introduced acetic acid in the Ni-CAT-1 and salicylic acid in the Co-CAT-1 synthesis (see Figure 2A). Top-view SEM images show a homogenous film and a long-range ordered array of pillar-like structures with a cross-section of 20–30 nm and evident gaps between the pillars of about 150 nm for both samples (see Figure 2B,C).

**Figure 3:**
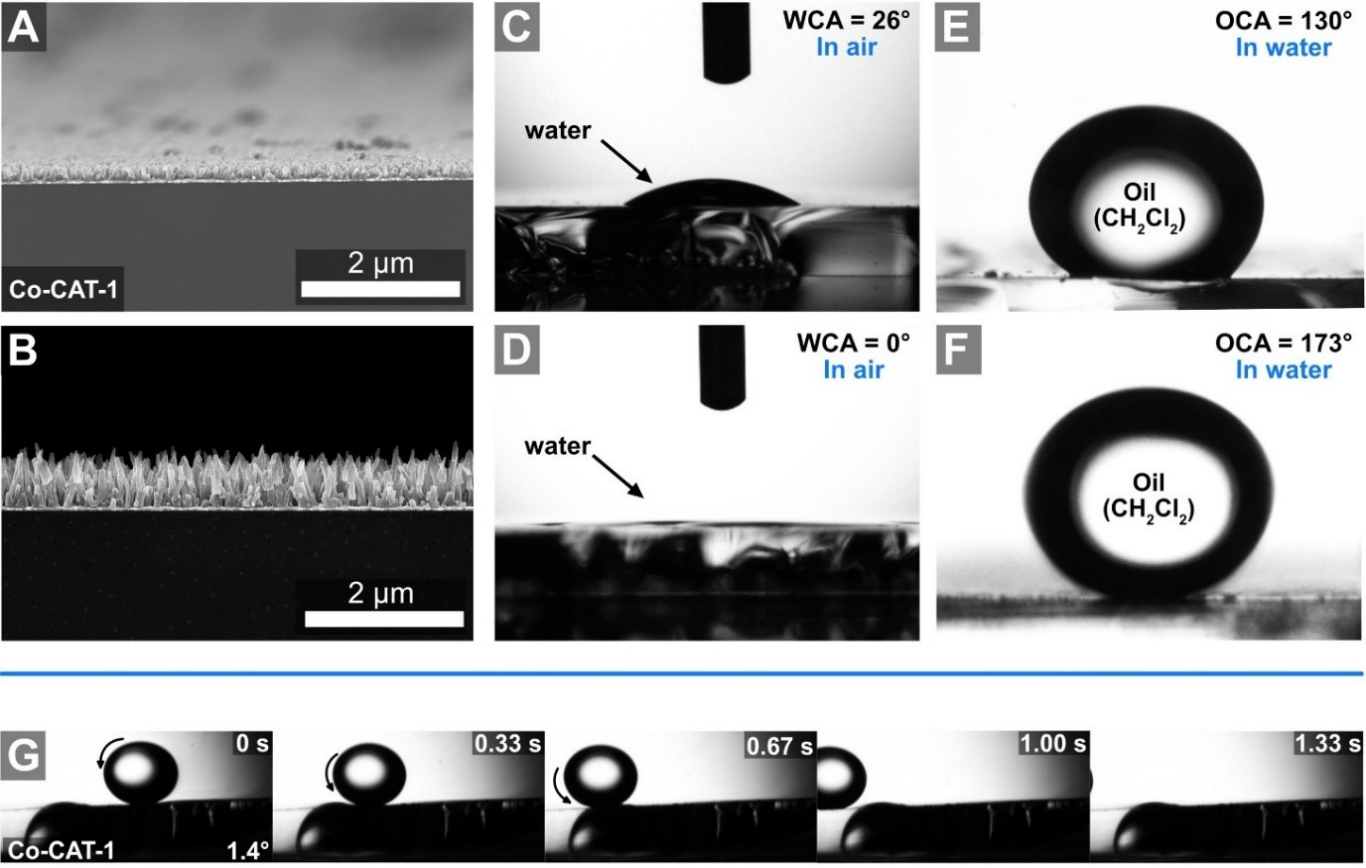
Cross-section SEM micrographs of A) an oriented and compact Co-CAT-1 film and B) an oriented (pillar-like) nanostructured Co-CAT-1 film. WCA of the C) compact (WCA = 26°) and the D) nanostructured (WCA = 0°) films. Underwater OCA (dichloromethane (DCM)) of the E) compact (CA = 130°) and the F) nanostructured films (CA = 173°). G) An image series of a self-cleaning surface experiment (3 frames per second) with DCM as an oil droplet placed on the nanostructured Co-CAT-1 film performed at a substrate tilt angle of 1.4°.

Cross-section SEM micrographs clearly visualize an array of vertically aligned MOF needles on the surface plane. In addition, a uniform film thickness of about 800 nm is observed throughout the cross-section of both Ni- and Co-CAT-1 samples, indicating a self-terminating growth under the employed conditions (Figure 3B and Figure S5.4, Supporting Information File 1). To confirm that the defined nanostructured film is indeed crystalline and the crystallites are well-aligned orthogonal to the surface plane, X-ray grazing incidence wide-angle scattering (GIWAXS) experiments were performed (see Figure 2B,C). GIWAXS analysis of the obtained films confirmed the successful on-surface deposition of crystalline M-CAT-1 materials. The collected out-of-plane reflections at *q*_y_ = 3.9 nm^−1^ (100), *q*_y_ = 7.1 nm^−1^ (200) and *q*_y_ = 9.2 nm^−1^ (210) near *q*_z_ = 0 were indexed according to the powder X-ray diffraction pattern of Co-CAT-1. The (004) reflection, referred to as the in-plane reflection, is visible as a diffuse arc at *q*_z_ =19 nm^−1^ (for further details see Supporting Information File 1). The latter corresponds to an interlayer distance of 3.3 Å. This points toward the preferential orientation of the M-CAT-1 crystals on the surface, where the crystallographic *c*-axis is positioned normal to the surface plane.

These data show that by employing the VAC synthesis approach in the presence of a specific crystallization promotor, the fabrication of crystalline nanostructured and oriented M-CAT-1 films on gold substrates is feasible. Deviating from these optimal conditions resulted in a poorly crystalline or inhomogeneous film (see Figure S5.10, Supporting Information File 1).

### Superhydrophilic and underwater superoleophobic MOF-based surfaces

Following Wenzels’ equation [70], an increased surface roughness results in a decreased WCA and in an enhanced OCA. To study the impact of roughness on the wetting properties of the M-CAT-1 films, we performed WCA and underwater OCA measurements for the compact and the nanostructured M-CAT-1 films (see Figure 3 and S5.5, Supporting Information File 1).

Oriented and compact films demonstrated CAs of 32° and 26° for Ni- and Co-CAT-1, respectively, whereas oriented and nanostructured films featured a drastic decrease in the WCA (see Figures S5.5, S5.7, Supporting Information File 1). Strikingly, we observed complete water spreading on the M-CAT-1 films and a WCA of 0° for both films, thus these surfaces can be considered superhydrophilic (Figure 3D and S5.5, Supporting Information File 1). These observed surface properties were further confirmed by measuring the underwater OCA with a droplet of dichloromethane (DCM). The oriented and compact films showed enhanced oleophobic properties with an underwater OCA of about 130° and 133° for Ni-CAT-1 and Co-CAT-1, respectively (see Figure 3 and S5.5, Supporting Information File 1). Remarkably, for the oriented and nanostructured films, we observed a strong increase in the OCA and extreme values of 174° and 173° were determined for Ni-CAT-1 and Co-CAT-1, respectively (see Figure S5.5, Supporting Information File 1). On these films, a nearly perfect oil sphere was formed, which is hardly in contact with the surface (see Figure S5.7, Supporting Information File 1). These surface properties resemble those of surface designs found in nature. For example, referring to marine life, fish scales in air show an amphiphilic behavior due to their high surface energy, but under water they turn superoleophobic, which is of great advantage for protection against hazardous environments, for example, oil-polluted water.

The self-cleaning properties of these extremely oleophobic M-CAT-1 nanostructured surfaces were demonstrated by casting a DCM oil droplet on a marginally tilted substrate (e.g., by 1.4°). Subsequently, the motion of the droplet was recorded by a high-speed camera with 3 frames per second (see Figure 3G and S5.6, Supporting Information File 1). The obtained image series reveals that the droplet crosses the substrate and rolls out of the detection range of the high-speed camera in about 1.3 s. According to the Cassi–Baxter theory, the combination of the very low tilt-angle that is required for the droplet to roll off the substrate and the extreme underwater OCA angle suggests that the DCM sphere is in contact only with the tips of the crystalline needles, whereas the intercrystallite areas only interact with water (Figure S7.1, Supporting Information File 1) [72]. To exclude the contribution of the gold surface to the wetting properties of the MOF films, we carried out control experiments with blank gold substrates giving a WCA of 94° and underwater OCA of 52° (see Figure S4.1, Supporting Information File 1). This confirms that the observed superhydrophilic and superoleophobic behavior arises from the MOF film.

To illustrate the stability of the MOF under harsh conditions, the films were subjected to an acidic environment (pH 5, 12 h), a basic environment (pH 9, 12 h), high temperature (150 °C, 12 h), and mechanical impact (sonication in water, 20 min). GIWAXS analysis in addition to WCA and OCA measurements after the respective treatment confirmed that the crystallinity, crystal orientation and extreme wetting properties were preserved (see Figures S8.1, S8.2, Supporting Information File 1).

**Figure 4:**
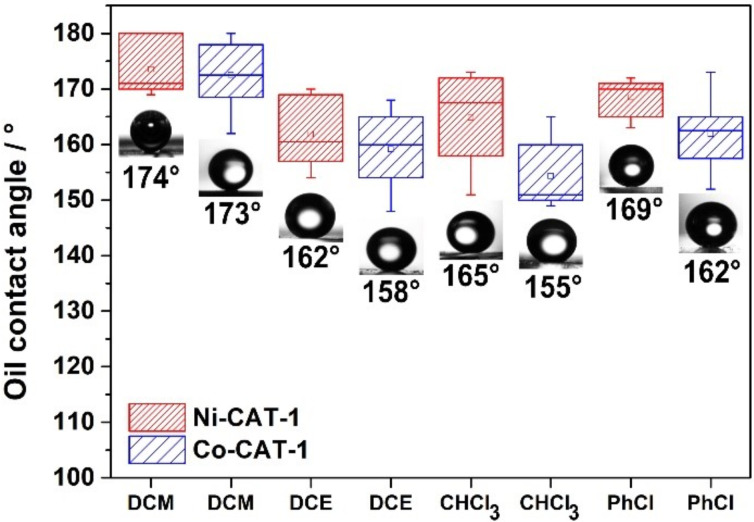
Box and whisker plot of the measured underwater OCAs for the nanostructured Ni-CAT-1 and Co-CAT-1 films. The employed liquids are dichloromethane (DCM), dichloroethane (DCE), chloroform (CHCl_3_) and chlorobenzene (PhCl). For all the tested liquids,a superoleophobicity is observed with underwater OCAs > 150°.

To establish these MOF films as superoleophobic surfaces applicable to a wide range of oils, we examined the interactions of other high-density oils with the oriented and nanostructured MOF surfaces. For this purpose, we determined the underwater OCA for different chlorinated liquids, such as dichloromethane (DCM), dichloroethane (DCE), chloroform (CHCl_3_) and chlorobenzene (PhCl). The OCAs were recorded using the exact same described procedure for DCM, and the results are plotted as box and whisker plots in Figure 4. For all the measured oils, superoleophobic behavior was observed with extreme OCAs ranging from 155° (DCE) for Co-CAT-1 to 169° (PhCl) for Ni-CAT-1 films. Specifically, the Ni-CAT-1 film exhibits the following OCAs: 174° (DCM), 162° (DCE), 165° (CHCl_3_) and 169° (PhCl), whereas the Co-CAT-1 film exhibits 173° (DCM), 158° (DCE), 155° (CHCl_3_) and 162° (PhCl). These results confirm that the MOF films efficiently repel a large variety of oily liquids, hence constituting an intriguing platform for underwater superoleophobic properties.

Since MOFs are crystalline materials featuring crystal facets with distinct surface energies, the orientation of these crystallites on a surface is expected to impact the wetting properties. In the following, we discuss the case of Ni-CAT-1 as a model for the M-CAT-1 series. In the context of crystal orientation on the surface, we analyzed the Ni-CAT-1 WCAs measured for pressed pellet samples and those of oriented and compact films. Interestingly, pressed pellet samples consisting of randomly distributed crystallites on the surface exhibit greater WCAs than oriented films, although the latter shows a slightly increased surface roughness (see Figures S5.2, S5.3, S3.4, S3.5, Supporting Information File 1). We attribute this difference to the different surface character which directly impacts the surface energy, and hence, the wetting properties (see Table S1, Supporting Information File 1). We therefore conclude that the observed wetting properties are due to a combination of two different significant contributions which can be controlled by the VAC process, namely the surface roughness and the crystallite orientation on the substrate.

### Antifog coating with MOF films

**Figure 5:**
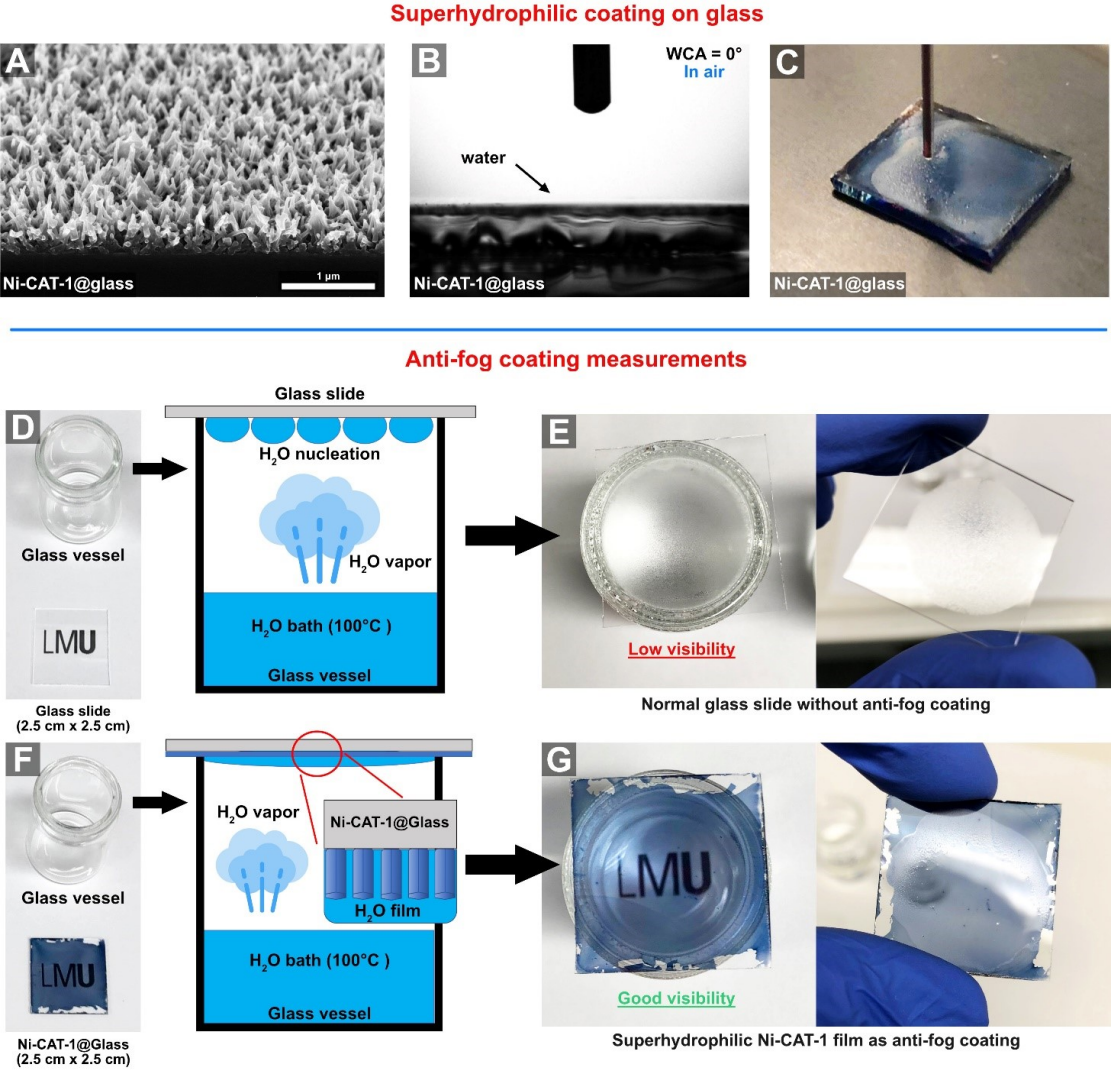
A) A 30° tilted SEM top view micrograph of Ni-CAT-1 film grown on glass. B) WCA on the superhydrophilic and nanostructured Ni-CAT-1 film. C) Macroscopic image of the Ni-CAT-1 on a glass substrate, showing a complete spreading of the water droplet. D) An antifog coating experiment performed with a bare glass slide that is placed above a glass vessel containing a reservoir of hot water at boiling temperature. E) The LMU seal obscured by water condensation on the bare glass. F) An antifog coating experiment performed with a MOF-coated glass above a glass vessel containing a reservoir of hot water at boiling temperature. G) High transparency and visibility through the substrate and a visible LMU seal is demonstrated.

Water condensation on surfaces is a known phenomenon occurring at the dew point on substrates such as glass, which serves as a favorable nucleation site for water droplets. This process leads to a substantial reduction in the transparency of the glass and the vision though the glass becomes extremely limited. To avoid condensation, modifications of the glass surface with different classes of functional coatings are necessary. Motivated by the superhydrophilic properties obtained by growing nanostructured M-CAT-1 films on gold substrates, we investigated the synthesis of such films as transparent coatings on glass substrates, thereby altering their wetting character towards superhydrophilic properties. Through this approach, a thin water layer decorating the textured MOF surface is expected to form, hindering the nucleation of water droplets on the glass surface. For this purpose, we employed the newly developed methodology for the synthesis of nanostructured Ni-CAT-1 films on gold substrates with a slightly modified synthesis procedure in order to address larger substrate dimensions such as 2.5 cm × 2.5 cm. The resulting MOF films on glass substrates reveal a blue-tinted, yet transparent coating (see Figure 5C,F). A top view SEM micrograph revealed the typical orthogonally aligned needle-like nanostructures on the surface, similar to the M-CAT-1 films grown on gold surfaces. The preferential orientation of the MOF crystals on the glass substrate was further confirmed by GIWAXS measurements (see Figure S5.8, Supporting Information File 1). The WCA measurements showed superhydrophilic properties similar to those observed for gold substrates (Figure 5C). Further control experiments confirmed that the superhydrophilic wetting properties originate from the presence of the nanostructured MOF film. Blank glass substrates showed WCA of 11° and OCA of 81° (see Figure S4.2, Supporting Information File 1). Furthermore, the MOF films endow the glass substrates with light-absorbing properties while maintaining transparency (see Figure S5.9, Supporting Information File 1). In addition, we found that these MOF films are stable in acidic (pH 5, 12 h) and basic conditions and at high temperature (150 °C, 12 h) (see Figure S8.3, Supporting Information File 1).

To test the antifog properties of the Ni-CAT-1 film, a glass vessel containing a reservoir of boiling water (≈100 °C) was placed above an LMU seal, and a non-modified glass substrate was positioned on top of the open vessel (see Figure 5D,F and S6.1, Supporting Information File 1). Within seconds, the seal was no longer visible, obscured by the water droplets generated by the condensation of steam on the glass surface (Figure 5E). In contrast, using a MOF-coated glass substrate, high transparency and clear visibility through the substrate were enabled, and the seal was visible even after longer exposure times (Figure 5F,G). To examine the behavior of the antifog coating under extreme temperature differences, the MOF-modified glass substrates were cooled down to −20 °C and subsequently placed on top of the hot water reservoir. Under all examined conditions, clear vision through the glass was maintained and water vapor nucleation was not observed (see Figure S6.2, Supporting Information File 1). This striking effect is attributed to the formation of a thin water layer on the MOF films which spreads out completely on the surface. This thin water layer is clearly visible by turning the substrate upside down (Figure 5G). This set of experiments establishes the pillar-like nanostructured Ni-CAT-1 films as highly efficient, antifog coatings featuring additional desired properties such as light absorbance. Furthermore, it underlines the power of VAC in enabling controlled bottom-up fabrication of MOF films with well-defined nanoscale morphologies and associated functionality.

## Conclusion

In this study, we demonstrated the controlled on-surface synthesis of crystalline, highly oriented and nanostructured M-CAT-1 films by employing a crystallization promoter in a VAC synthesis. First, the free surface energy of M-CAT-1 was calculated for pressed pellet samples, shedding light on the amphiphilic wetting properties (both hydrophilic and oleophilic) of the materials in air. Oriented and compact M-CAT-1 films exhibited enhanced wetting properties where shallower contact angles were deter-mined for water (about 32°) and a large underwater OCA (130°), indicating oleophobic properties. The corresponding oriented and nanostructured M-CAT-1 films exhibited extreme wetting properties with a WCA of 0° and an underwater OCA of 174°, and are thus demonstrated as being superhydrophilic and superoleophobic in water.

The underwater self-cleaning ability of these films was demonstrated by depositing an oil droplet on a substrate tilted at a shallow angle of 1.4°. The effective underwater oil-repelling properties of the M-CAT-1 films were confirmed by employing a variety of different chlorinated oily liquids. Under ambient atmosphere, the water wetting properties of these systems could be drastically altered by changing the film morphology from compact to pillar-like nanostructures. This effect was also transferred onto transparent substrates such as glass, resulting in an efficient, transparent, MOF-based, antifog coating. Hence, we demonstrate that on-surface alteration of the MOF film morphology by versatile solution-based bottom-up methods such as VAC is a powerful tool for realizing the potential of MOFs in surface-based technologies such as oil–water separation systems, antioil coatings, or self-cleaning surfaces. Furthermore, the use of a MOF allows for encoding further desired surface properties such as light absorption and electrical conductivity. This can be valuable for applications such as photo-electrocatalysis water splitting where a high textural surface area, water contact and light absorption are required.

## Supporting Information

File 1 Additional experimental details.
