# Supplementary material for: Nanostructured and oriented metal–organic framework films enabling extreme surface wetting properties
Source: Beilstein J Nanotechnol. 2019 Oct 9;10:1994–2003. doi: 10.3762/bjnano.10.196 (PMC6808203; doi:10.3762/bjnano.10.196)
Supplement: File 1 — Additional experimental details. [file Beilstein_J_Nanotechnol-10-1994-s001.pdf]

BEILSTEIN JOURNAL OF NANOTECHNOLOGY

Supporting Information

for

Nanostructured and oriented metal–organic framework films enabling extreme surface wetting properties

Andre Mähringer, Julian M. Rotter and Dana D. Medina

Beilstein J. Nanotechnol. **2019,** *10,* 1994–2003. doi:10.3762/bjnano.10.196

Additional experimental details

License and Terms: This is a supporting information file under the terms of the Creative Commons Attribution License (http://creativecommons.org/licenses/by/4.0). Please note that the reuse, redistribution and reproduction in particular requires that the authors and source are credited.
The license is subject to the *Beilstein Journal of Nanotechnology* terms and conditions: (https://www.beilstein-journals.org/bjnano)

Table of Contents

1. Characterization S3

X-ray spectroscopy S3

Scanning electron microscopy (SEM) S3

Contact angle measurements S3

Atomic force microscopy (AFM) S3

2. Experimental S4

General S4

Substrates S4

Bulk Synthesis S4

Ni-CAT-1 .... S4

Co-CAT-1 .... S5

Preparation of Ni- and Co-CAT-1 pellets S5

Film formation via vapor-assisted conversion (VAC) S5

Synthesis of compact Ni- and Co-CAT-1 films on gold surfaces S5

Synthesis of nanostructured Ni-CAT-1 films on gold substrates S6

Synthesis of nanostructured Co-CAT-1 films on gold substrates S6

Synthesis of nanostructured Ni-CAT-1 films on glass substrates S7

3. Characterization of the M-CAT-1 bulk material S8

PXRD and SEM analysis of Ni- and Co-CAT-1 bulk materials S8

PXRD and SEM analysis of pelletized Ni- and Co-CAT-1 samples S8

Water and Oil-contact angles of Ni- and Co-CAT-1 pellets in water S9

Atomic force microscopy (AFM) of M-CAT-1 Pellets S9

Surface energy of M-CATs S10

4. Reference contact angle measurements S13

Contact Angle (CA) S13

CA measurements on gold substrates S13

CA measurements on glass substrates S14

5. Characterization of M-CAT-1 thin films S15

GIWAXS and SEM of oriented and compact Ni- and Co-CAT-1 thin films S15

AFM of oriented and compact Ni- and Co-CAT thin films S16

Nanostructured Ni- and Co-CAT-1 thin films on gold substrates S17

Water and oil-contact angles of compact and oriented Ni-CAT-1 films S17
Photographs of the WCA and OCA measurements S18
GIWAXS and SEM analysis of nanostructured films grown on glass substrates S18
UV-vis of nanostructured Ni-CAT-1 films grown on quartz S19
Impact of precursor and crystallization promotor concentration on the film growth S19

6 Anti-fog coating experiments S20
Macroscopic photograph of the anti-fog coating experiment S20
Temperature-dependent anti-fog coating experiments S20

7 Wetting models S21

8 Stability Test S22
Ni-CAT-1 oriented and nanostructured films on gold substrates S22
Co-CAT-1 oriented and nanostructured films on gold substrates S23
Nanostructured Ni-CAT-1 films on glass substrates S24

References S24

1. Characterization

X-ray spectroscopy

X-ray diffraction (XRD) analyses were performed on a *Bruker D8* diffractometer in Bragg-Brentano geometry with Ni-filtered Cu K_{α} (λ = 1.54060 Å) radiation operating at 40 kV and 30 mA with a position-sensitive detector (*LynxEye*).

Two-dimensional grazing-incident wide angle X-ray scattering (2D GIWAXS) data were collected using an *Anton-Paar SAXSpace system* equipped with a Cu K_{α} microfocus source operated at 50 kV and 1 mA and an *Eiger Dectris R 1M* 2D detector.

Scanning electron microscopy (SEM)

SEM images were recorded on a *FEI Helios NanoLab G3 UC* electron microscope with an acceleration voltage of 2 kV from a field emission gun. For the cross-section analysis substrates were partially cut and broken manually to reveal fresh cross-sections. Prior to SEM analysis the samples were coated with a thin carbon layer by carbon fiber flash evaporation in high vacuum.

Contact angle measurements

Contact-angle measurements were performed on an *attension* from *Biolin Scientific*. A droplet with the sessile drop method. A droplet of liquid was placed on the substrate, while recording images with 1 fps for 10s, while the dynamic measurements (self-cleaning surfaces) were recorded with 3 fps for 10 s. The image analysis was carried out with the software *ImageJ* using the manual mode and fitting the contour of the droplets manually.

Atomic force microscopy (AFM)

AFM measurements were performed using a *NANOINK* atomic force microscope in tapping mode with a scanning rate of 0.2 Hz, a proportional gain of 50 and an integral gain of 32. The scanned area was 5.02 × 5.02 µm at 282 × 282 pixels.

2. Experimental

General

All materials were purchased from *Sigma Aldrich, Acros or TCI Europe* in the common purities *purum*, *puriss* or *reagent grade.* The materials were used as received without additional purification and handled in air unless otherwise noted.

The water utilized in the synthesis was subjected to a *Merck-Milipore Mili-Q* purification system prior to use.

Substrates

Analogous as described in [S1]. The preparation of the gold substrates was based on the procedure described by Hinterholzinger *et al*. Seven microscope glass slides (*Menzel*, 76 mm x 26 mm) in a support made of Teflon were cleaned by ultrasonic treatment in acetone, followed by sequential washing steps with 2-propanol, a 1:100 mixture of *Hellmanex III* and water, and finally 2-propanol (*Chromasolv*). Oxygen plasma cleaning (*Diener electronic*, Plasma-Surface-Technology) for 30 min was conducted, previous to the mounting of the glass slides in a vacuum deposition unit installed in a glove box (*MBraun Labmaster Pro SP* equipped with an *Inficon SQC-310C Deposition controller*). 10 nm of titanium and 40 nm of gold were thermally deposited under high vacuum onto the microscope glass slides.

The respective substrates were prepared by cutting commercially available microscope slides (*Menzel*, 76 mm x 26 mm), gold coated glass slides into pieces of 1.2 cm x 1.0 cm and 2.5 cm x 2.5 cm.

Bulk Synthesis

Ni-CAT-1

Analogous as described in [S1]. In a 5 mL culture tube, a solid mixture of 2,3,6,7,10,11-hexahydroxytriphenylene (0.0107 mmol, 3.5 mg) and nickel(II) acetate tetrahydrate (0.0201 mmol, 5 mg) was suspended in 1 mL 1-propanol and 1 mL distilled water (1:1 v:v). The loading of the culture tube was carried out under ambient atmosphere and the resulting blue solution was sonicated for 10 s at maximum power. Subsequently, the culture tube was tightly capped and heated at 85 °C for 18 h in a preheated oven. The resulting black solution was washed

three times with 10 mL dry acetone and isolated by centrifugation. The obtained dark blue microcrystalline product was dried under dynamic vacuum prior to analysis.

Co-CAT-1

Analogous as described in [S1]. In a 5 mL culture tube, a solid mixture of 2,3,6,7,10,11-hexahydroxytriphenylene (0.0107 mmol, 3.5 mg) and cobalt(II) acetate tetrahydrate (0.0207 mmol, 5 mg) was suspended in 1 mL of 1-propanol and 1 mL of distilled water (1:1 v:v). The loading of the culture tube was carried out under ambient atmosphere and the resulting blue solution was sonicated for 10 s at maximum power. Subsequently, the culture tube was tightly capped and heated at 85 °C for 18 h in a preheated oven. The resulting black solution was washed three times with 10 mL of dry acetone and isolated by centrifugation. The obtained dark blue microcrystalline product was dried under dynamic vacuum prior to analysis.

Preparation of Ni- and Co-CAT-1 pellets

Analogous as described in [S1]. Ni- (110.5 mg) and Co- (95.8 mg) CAT-1 pellets (obtained from several of the above described batches) for electrical conductivity measurements were fabricated with a standard Paul-Weber KBr Press.

Film formation via vapor-assisted conversion (VAC)

Analogous as described in [S1]. For the film formation using VAC, a glass bottle (*Schott Duran*, borosilicate 3.3, *ISO4796*, 100 mL) with a *PBT* cap equipped with a Teflon seal was used. The bottom part of the bottle was filled with 14 *Raschig*-rings (10 mm x 10 mm, soda-lime glass) to obtain an elevated flat platform for the substrate. A mixture of water and 1-propanol was filled into the bottle. Afterwards, a substrate (1.2 cm x 1 cm) was placed on top of the *Raschig*-rings and fully coated with a liquid layer of a freshly prepared MOF precursor solution. The bottle was closed and transferred into a preheated oven where it was kept for the specified time. Afterwards, the autoclave was removed from the oven and cooled down for 10 min before the substrate was rinsed with dry acetone.

Synthesis of compact Ni- and Co-CAT-1 films on gold surfaces

Analogous as described in [S1]. The HHTP precursor (2.69 x 10^{-3} mmol; 0.875 mg) and the metal precursors nickel acetate (5.0 x 10^{-3} mmol; 1.25 mg), cobalt acetate (5.0 x 10^{-3} mmol; 1.25 mg) or copper trifluoroacetylacetonate (5.0 x 10^{-3} mmol; 1.25 mg) were mixed in a

defined ratio (1:2) and dissolved in a solvent mixture of water and 1-propanol (1.5 mL : 1.5 mL) by sonication for 20 s at maximum power. The obtained precursor solution was filtered through a syringe filter to remove any remaining particles. Subsequent, 40 µL of the solution (HHTP: 0.899 mmol L^{-1}; MOAc: 1.674 mmol L^{-1}) were deposited onto a clean 1.2 x 1.0 cm gold substrate. Four of these set-ups were placed onto glass spacers in an autoclave loaded with a solvent mixture of water and 1-propanol (2.5 mL : 2.5 mL). The autoclave was sealed immediately and placed into a preheated oven at 85 °C for 3.5 h. The experimental set-up of the VAC is shown in Fig. S2.1. Afterwards, the autoclave was removed from the oven and cooled down for 10 min before the substrate was rinsed with dry acetone.

Synthesis of nanostructured Ni-CAT-1 films on gold substrates

The precursor solution was prepared by providing a hexahydroxytriphenylene (HHTP) precursor (5.4 x 10^{-3} mmol; 1.75 mg) as the organic building blocks and nickel acetate (10 x 10^{-3} mmol; 2.5 mg) as the metallic precursor in a defined ratio (1:2 n:n) and dissolving them in a solvent mixture of water and 1-propanol (1 mL : 1 mL) with the addition of a modulator acetic acid (50 µL). 40 µL of the respective precursor solution (HHTP: 2.75 mmol L^{-1}; NiAc: 5 mmol L^{-1}; HOAc: 0.41 mol L^{-1}) was dropcasted onto the surface of a clean 1.2 x 1.0 cm gold coated glass substrate. Four of such substrates, each carrying a thin layer of precursor solution, were placed onto glass spacers as substrate holders in an autoclave partly filled with a solvent bath consisting of water and 1-propanol (2.5 mL : 2.5 mL). The autoclave was sealed immediately and placed into a preheated oven at 85°C for 12 hours. Afterwards, the autoclave was removed from the oven and cooled down for 10 min, before the substrates were rinsed with dry acetone.

Synthesis of nanostructured Co-CAT-1 films on gold substrates

The precursor solution was prepared by providing an HHTP precursor (5.4 x 10^{-3} mmol; 1.75 mg) as the organic building blocks and cobalt acetate (11.0 x 10^{-3} mmol; 3 mg) as the metallic precursors in a defined ratio (1:2 n:n) and dissolving them in a solvent mixture of water, 1-propanol (1 mL : 1 mL) with addition of 50 µL of a water, 1-propanol solution (1 mL : 1 mL) containing salicylic acid as a modulator (0.89 mmol; 125 mg). 40 uL of the respective precursor solution (HHTP: 2.75 mmol L^{-1}; CoAc: 6 mmol L^{-1}; salicylic acid: 0.01 mmol L^{-1}) was dropcasted onto a clean 1.2 x 1.0 cm gold coated substrate. Four of such substrates, each carrying a thin

layer of precursor solution, were placed onto glass spacers as substrate holders in an autoclave partly filled with a solvent bath consisting of water and 1-propanol (2.5 mL : 2.5 mL). The autoclave was sealed immediately and placed into a preheated oven at 85°C for 12 h. Afterwards, the autoclave was removed from the oven and cooled down for 10 min, before the substrates were rinsed with dry acetone.

Synthesis of nanostructured Ni-CAT-1 films on glass substrates

Analogous as described in [S1]. The HHTP precursor (5.4 x 10^{-3} mmol; 1.75 mg) and the nickel acetate (10.0 x 10^{-3} mmol; 2.5 mg) were mixed in a defined ratio (1:2) and dissolved in a solvent mixture of water and 1-propanol (1 mL : 1 mL) with the addition of the modulator acetic acid (50 µL). 100 µL of the respective precursor solution (HHTP: 2.75 mmol L^{-1}; NiAc: 6 mmol L^{-1}; HOAc: 0.41 mol L^{-1}) were deposited onto a clean 2.5 x 2.5 cm glass substrate. One of this set-ups was placed onto glass spacers in an autoclave loaded with a solvent mixture of water and 1-propanol (2.5 mL : 2.5 mL). The autoclave was sealed immediately and placed into a preheated oven at 85°C for 18 h. Afterwards, the autoclave was removed from the oven and cooled down for 10 min before the substrates were rinsed with dry acetone.

3. Characterization of the M-CAT-1 bulk material

PXRD and SEM analysis of Ni- and Co-CAT-1 bulk materials

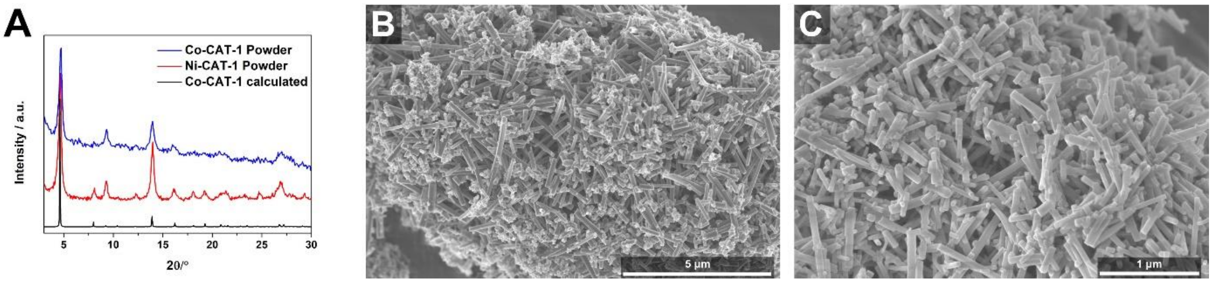

Figure S3.1 A) PXRD pattern of the Ni- and Co-CAT-1 (red and blue) powders compared to the calculated Co-CAT-1 pattern (black)**. B)** and **C)** show the needle-like bulk morphology of the Ni- and Co-CAT-1 crystal, respectively.

PXRD and SEM analysis of pelletized Ni- and Co-CAT-1 samples

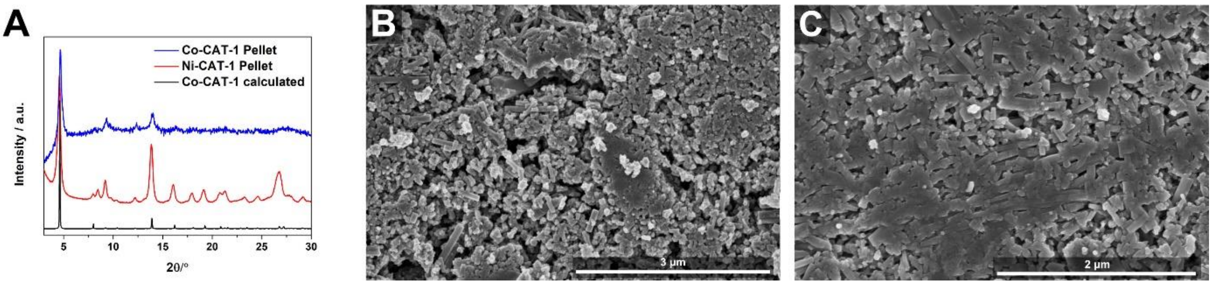

Figure S3.2 A) PXRD pattern of the Ni- and Co-CAT-1 (red and blue) pelletized materials compared to the calculated Co-CAT-1 pattern (black). **B)** and **C)** show the dense needle-like bulk surface of the Ni- and Co-CAT-1 pelletized samples, respectively.

Water and Oil-contact angles of Ni- and Co-CAT-1 pellets in water

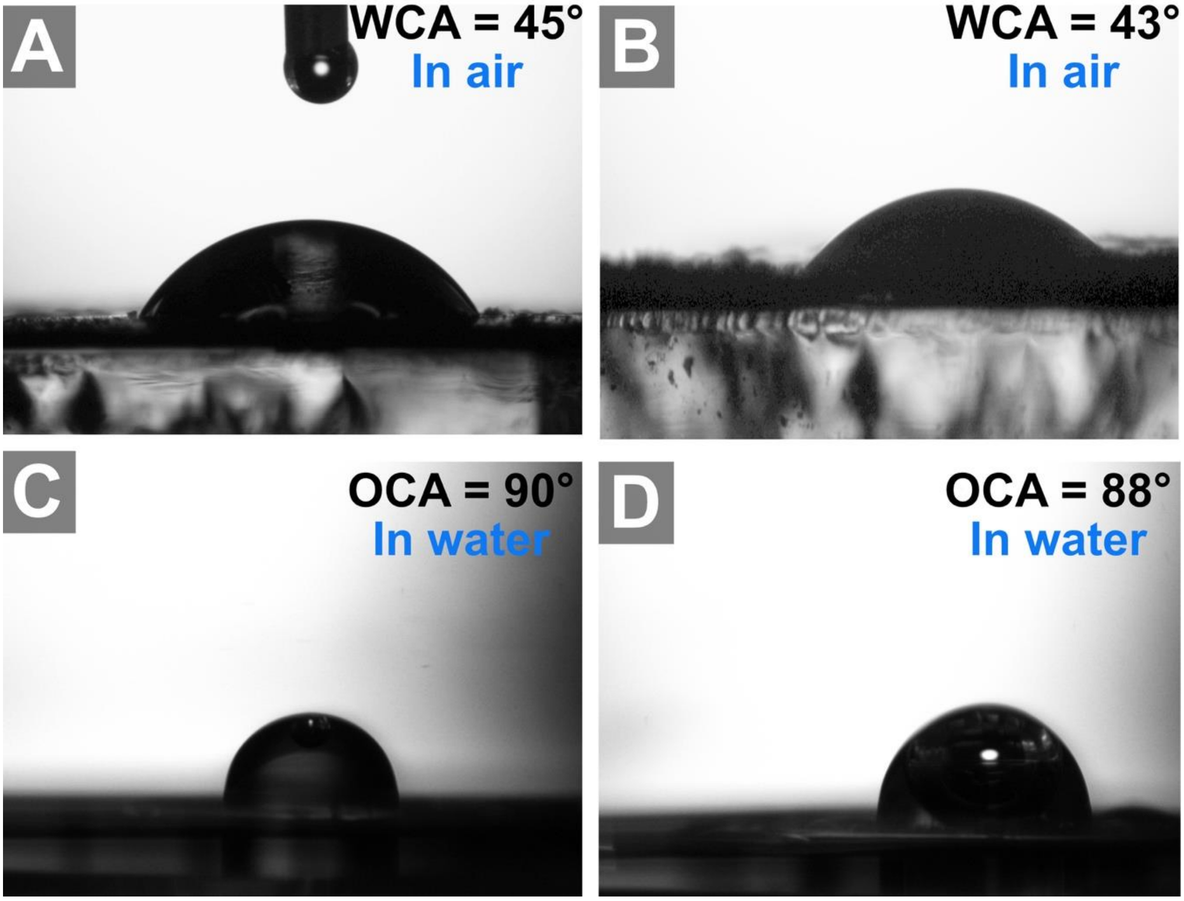

Figure S3.3 A) and **B)** Water contact angle of Ni- and Co-CAT-1 pellet samples in air, respectively. **C)** and **D)** underwater dichloromethane contact angle of Ni- and Co-CAT-1 pellet samples, respectively.

Atomic force microscopy (AFM) of M-CAT-1 Pellets

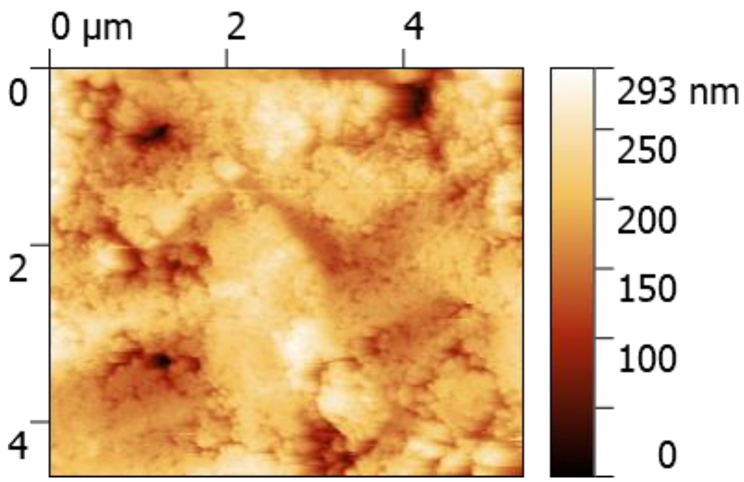

Figure S3.4 AFM topographic measurement of a Ni-CAT-1 pelletized sample (4.5 µm x 4.7 µm). The surface roughness values were calculated to be 37 nm (R_{ms}).

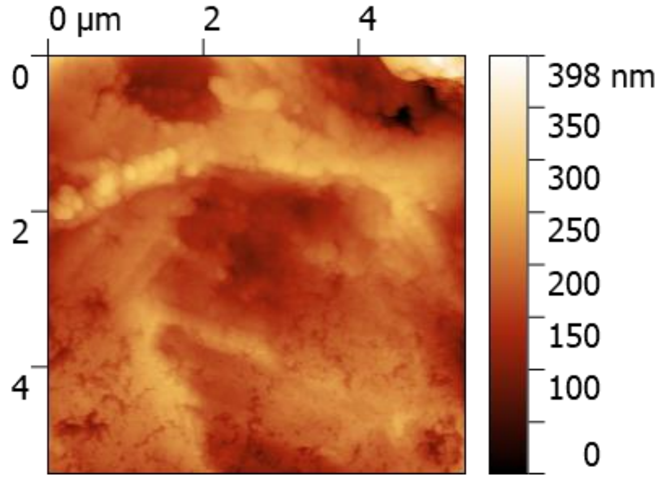

Figure S3.5 AFM topographic measurement of a Co-CAT-1 pelletized sample (4.5 µm x 4.7 µm). The surface roughness values were calculated to be 44 nm (R_{ms}).

Surface energy of M-CATs

The wetting properties of the M-CAT-1 were estimated by the following procedure. First, M-CAT-1 bulk materials were prepared according to a literature procedure. Subsequently, the obtained microcrystalline powders were pressed to pelletized samples (1 cm diameter, thicknesses 500 µm; 100 mg material). The samples exhibit an even surface composed of densely packed and randomly oriented crystallites. This was confirmed by scanning-electron microscopy (SEM) and X-ray diffraction (XRD) analysis and atomic force microscopy (AFM). On the as-prepared pelletized samples we performed contact angle (CA) measurements to estimate the free solid surface energy, especially the dispersive and polar components. The free surface energy of a solid cannot be directly measured, but it can be calculated from a set of steady state liquid/solid contact angles. Therefore, we used a common method for estimating the free surface energy, the *Fowkes theory*. This theory describes the surface energy of a solid as composed of a dispersive component, and “non”-dispersive part (polar). It is therefore based on the *Young’s* equation and the *Dupre’s* definition of adhesion energy showing that the adhesive energy between a solid and a liquid can be separated into dispersive interactions and the non-dispersive (polar) interactions between the two phases.

These two energy expressions *e.g. Young’s* and the *Dupre’s* equations can be combined to yield the *Fowkes’* surface energy equation:

$$(\sigma_L^D)^{1/2}(\sigma_S^D)^{1/2} + (\sigma_L^P)^{1/2}(\sigma_S^P)^{1/2} = \frac{\sigma_L(cos\theta + 1)}{2} \quad (1)$$

with $\sigma_L{}^D$ =surface tension liquid (dispersive), $\sigma_L{}^P$ = surface tension liquid (polar), $\sigma_S{}^D$ = surface free energy solid (dispersive), $\sigma_S{}^P$ = free surface energy of the solid (polar), σ_L = overall surface tension liquid, ϑ = measured contact angle at the liquid/solid interface. In order to determine the surface energy, we tested the solid surface using a liquid which exhibits only a dispersive component to its overall surface tension (σ_L = $\sigma_L{}^D$ = 50.8 mN/m) featuring a high surface tension, hence diiodmethane ($\sigma_L{}^P$=0) was the solvent of choice. Straightforwardly, eq. (1) can be expressed as:

$$\sigma_S^D = \frac{\sigma_L(cos\theta + 1)^2}{4} \quad (2)$$

$\sigma_S{}^D$ can be directly determined from the measured CA of diiodmethane. In the next step, we proceeded with measuring the CA of distilled water showing one polar and one dispersive component to its overall surface tension ($\sigma_L{}^P$ = 46.4 mN/m, and $\sigma_L{}^D$ = 26.4 mN/m). Knowing these components, we calculated $\sigma_S{}^P$ as the only unknown paramenter in eq. (1). By adding the dispersive and the polar components of the solid, we finally obtained the σ_S overall surface energy of the M-CAT-1 materials (equation 3).

$$\sigma_S^D + \sigma_S^P = \sigma_S \quad (3)$$

Table S1. Overall surface energies (σ_S) and the respective dispersive (σ_S^D) and polar component (σ_S^P) estimated for Ni- and Co-CAT-1 oriented and compact films (OCF) and oriented and nanostructured films (ONF).

M-CAT-1	σ_s [mN / m]	σ_s^D [mN/m]	σ_s^P [mN/m]
Ni-CAT-1(OCF)	71.3	50.8	20.4
Co-CAT-1(OCF)	73.6	50.8	22.8
Ni-CAT-1(ONF)	79.5	50.8	28.5
Co-CAT-1(ONF)	79.5	50.8	28.5

4. Reference contact angle measurements

Contact Angle (CA)

The contact angle (CA) describes the measured angle between a liquid-vapor interface in direct contact with a solid surface. From the obtained CA the wettability of the solid surface can be analyzed.

CA measurements on gold substrates

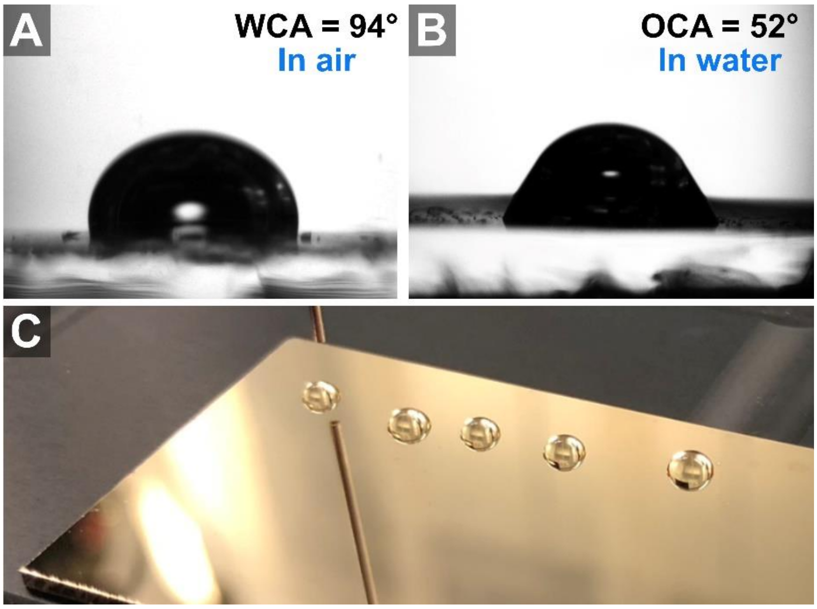

Figure S4.1 A) CA measurements of a bare gold surface (right after deposition) in air. **B)** OCA measurements of a bare gold surface in water with dichloromethane as an oil test liquid **C)** Macroscopic photographs of water droplets on a bare gold surface used for WCA measurements in air.

CA measurements on glass substrates

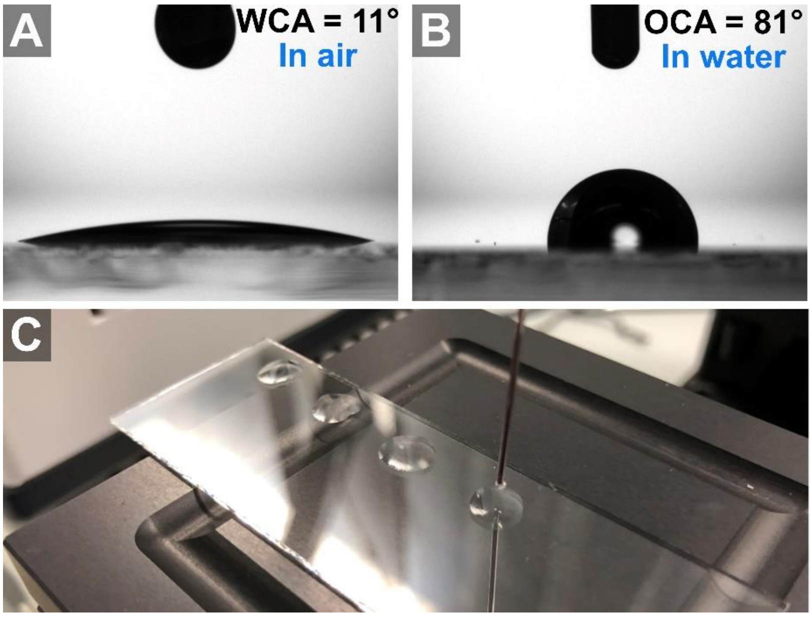

Figure S4.2 A) WCA on a bare glass surface in air. **B)** Under water OCA of dichloromethane droplet on a bare glass surface **C)** Macroscopic photographs of water droplets placed on a bare glass surface in air.

5. Characterization of M-CAT-1 thin films

GIWAXS and SEM of oriented and compact Ni- and Co-CAT-1 thin films

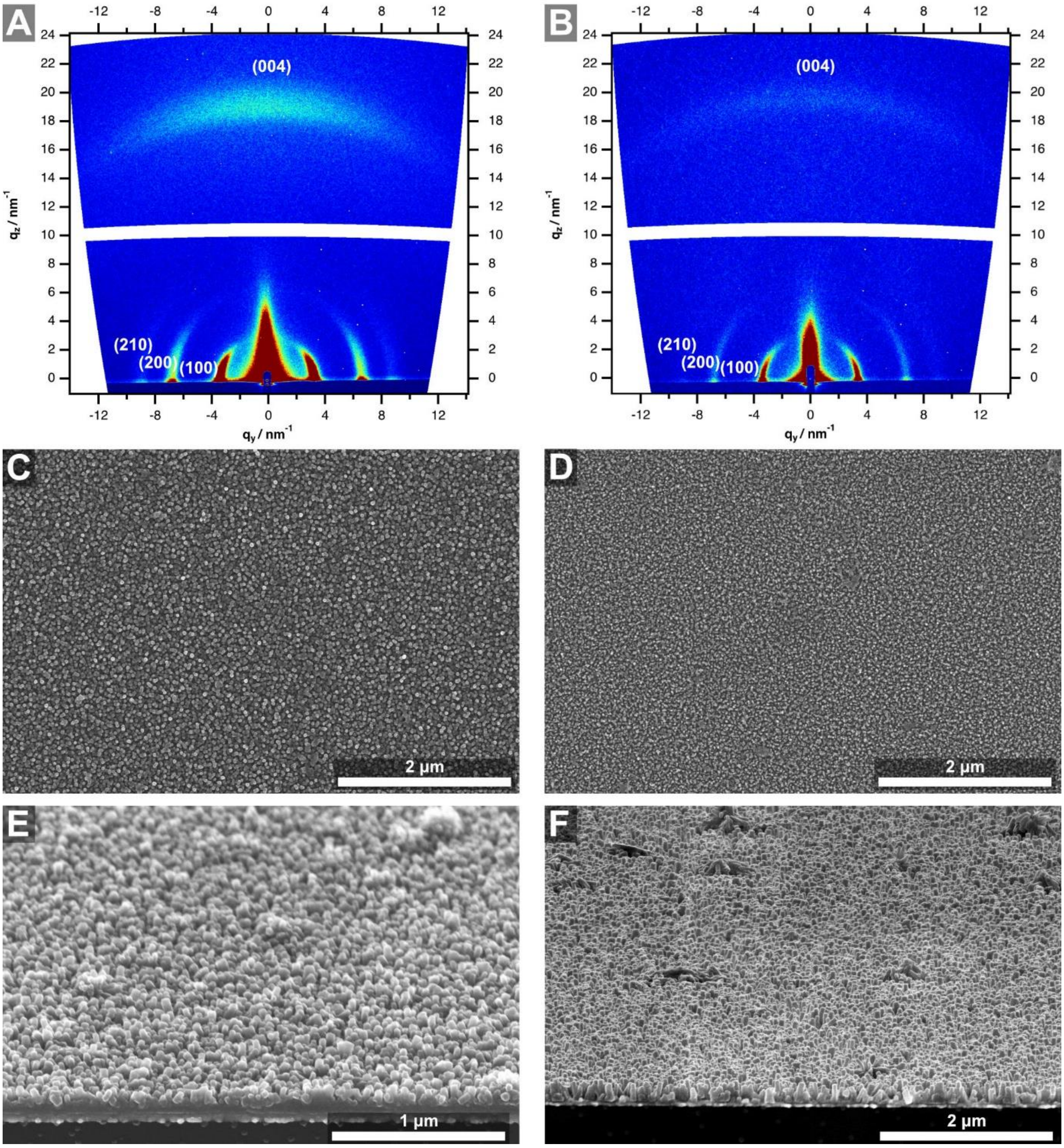

Figure S5.1 A), C) and **E**) Depict a the GIWAXS measurement of an oriented and compact Ni-CAT-1 film on gold, top-view SEM and 30° tilted SEM cross-section images of the same sample, respectively. **B), D)** and **F**) Depict a GIWAXS measurement of an oriented and compact Co-CAT-1 film on gold, top-view SEM and 30° tilted SEM cross-section images of the same sample, respectively

AFM of oriented and compact Ni- and Co-CAT thin films

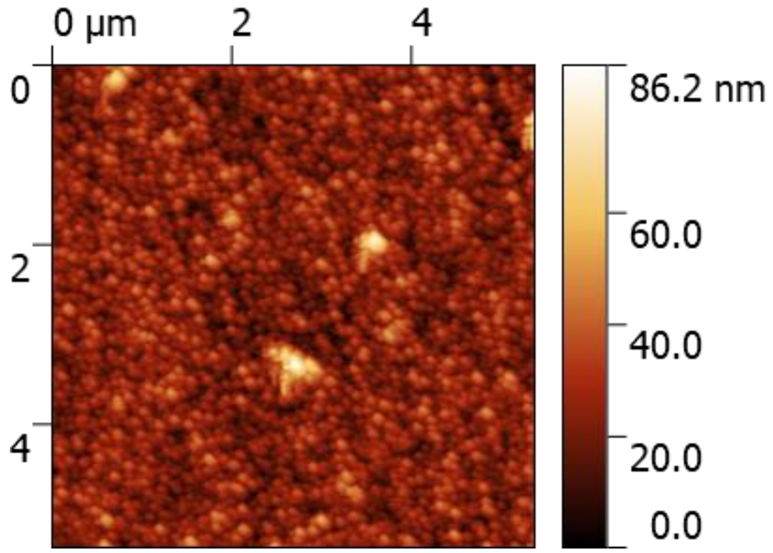

Figure S5.2 Atomic force microscopy topographic image of a of an oriented and compact Ni-CAT-1 thin film (4.5 µm x 4.7 µm area). Measured roughness (Rms) of 10.2 nm.

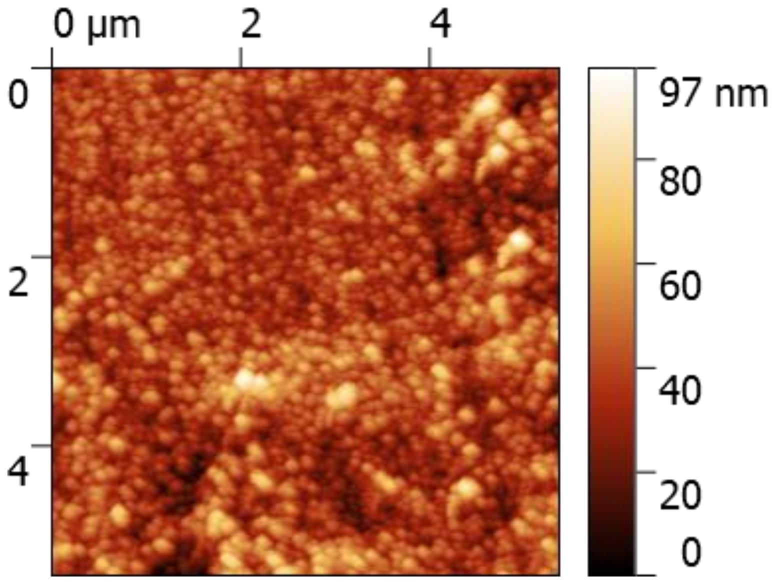

Figure S5.3. Atomic force microscopy topographic image of a area of an oriented and compact Co-CAT-1 film (4.5 µm x 4.7 µm). Measured roughness Rms of 14.1 nm.

Nanostructured Ni- and Co-CAT-1 thin films on gold substrates

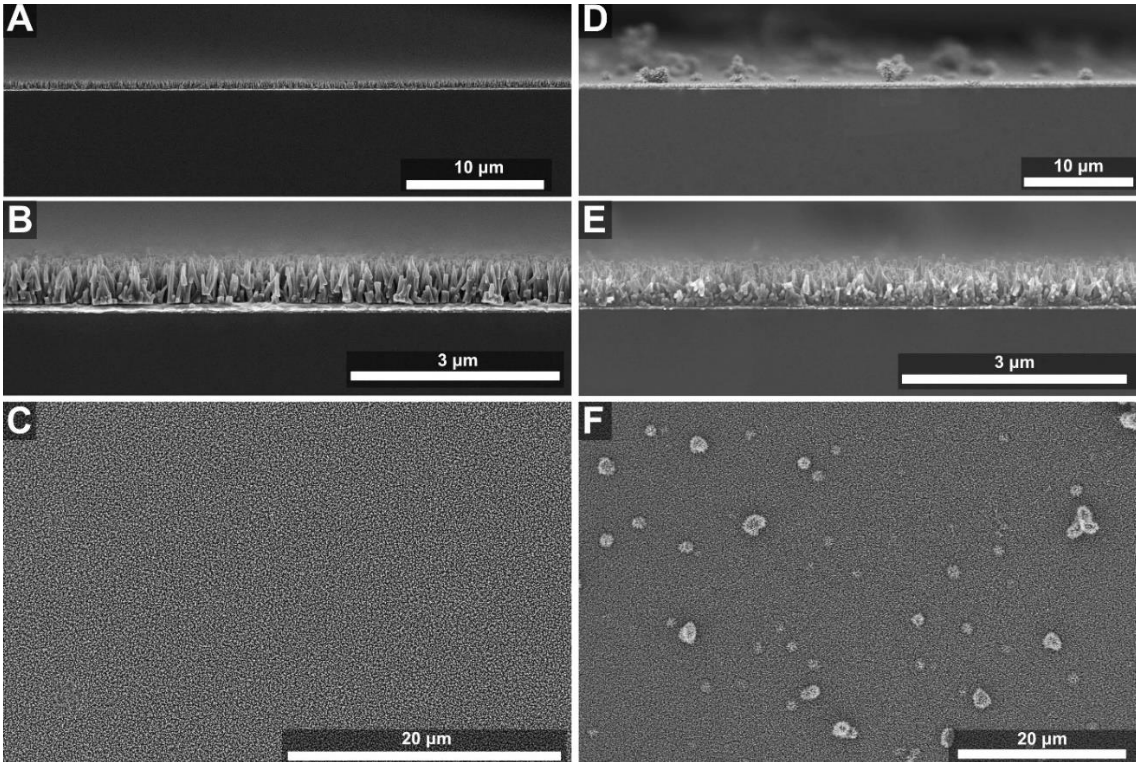

Figure S5.4 A), B) and **C**) Depict the cross-section, a magnified cross-section image, and a top-view micrograph of Co-CAT-1 grown on gold as a nanostructured film, respectively. **D), E)** and **F**) Depict the cross-section, a magnified cross-section image, and a top-view micrograph of Ni-CAT-1 grown on gold as a nanostructured film, respectively.

Water and oil-contact angles of compact and oriented Ni-CAT-1 films

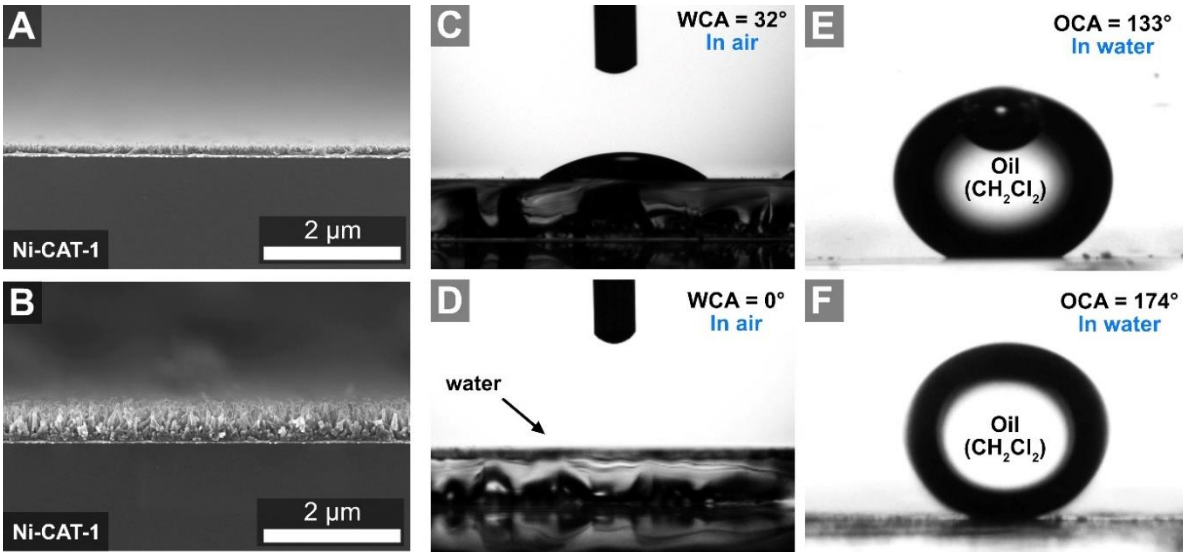

Figure S5.5 Cross-section SEM micrographs of A) an oriented and compact Ni-CAT-1 film and B) an oriented (pillar-like) nanostructured Ni-CAT-1 film. C) and D) WCA obtained for the compact (WCA = 32°) and the nanostructured (WCA = 0°) films, respectively. E) and F) Underwater OCA obtained for the compact (CA = 133°) and the nanostructured film (CA = 173°) with dichloromethane (DCM), respectively.

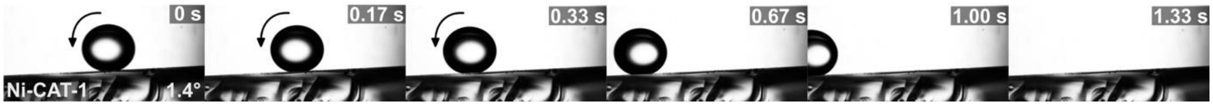

Figure S5.6 An image series of a self-cleaning surface experiment (3 fps) with DCM as an oil droplet placed on a tilted nanostructured Ni-CAT-1 film (tilt angle of 1.4°).

Photographs of the WCA and OCA measurements

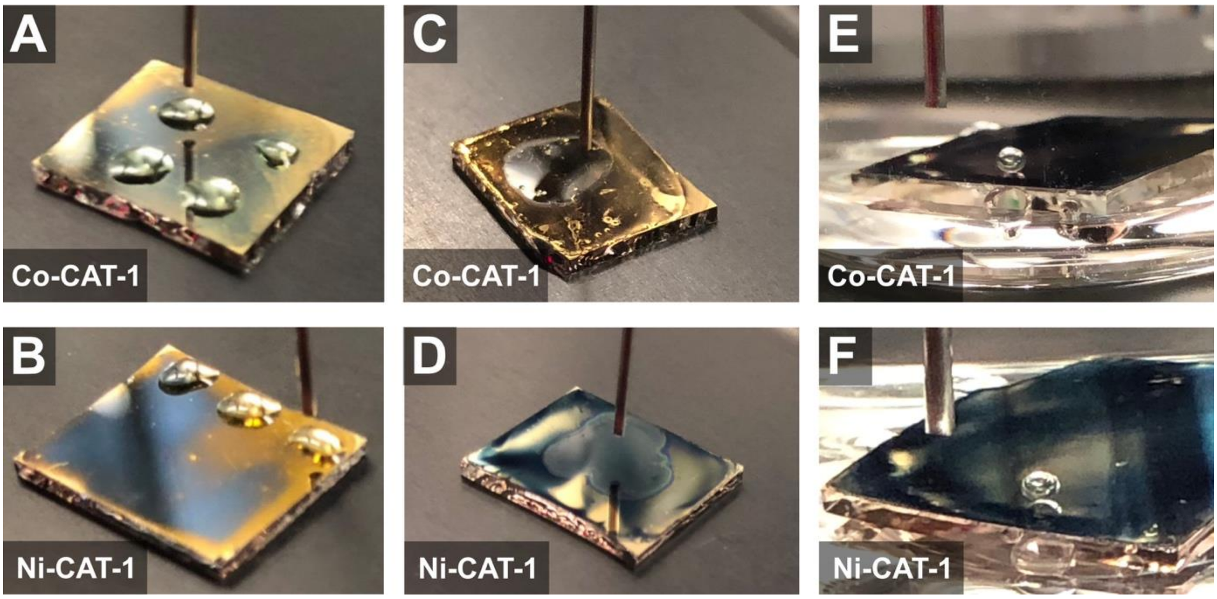

Figure S5.7 A) and **B)** Macroscopic macrographs of the WCA experiments on compact Co- and Ni-CAT-1 films. **C)** and **D)** WCA experiments on nanostructured Co- and Ni-CAT-1 films in water. **E)** and **F)** OCA experiments on nanostructured Co- and Ni-CAT-1 films in water.

GIWAXS and SEM analysis of nanostructured films grown on glass substrates

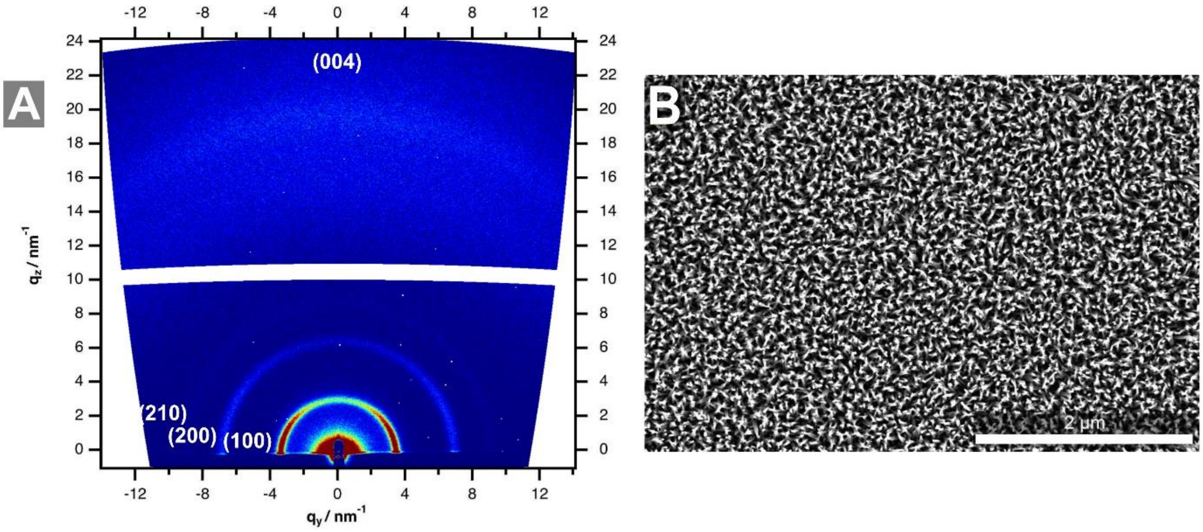

Figure S5.8 A) GIWAXS pattern of a Ni-CAT-1 film featuring preferred crystal orientation on a glass substrate**. B**) Corresponding top-view SEM micrograph of the film**.**

UV-vis of nanostructured Ni-CAT-1 films grown on quartz

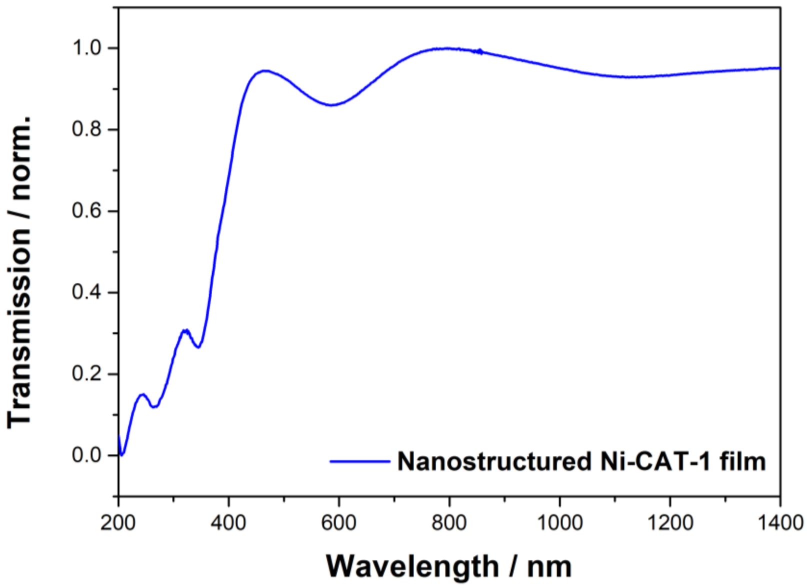

Figure S5.9 Transmission UV-vis spectrum of Ni-CAT-1 film grown on a quartz substrate. The material exhibits a strong absorbance with an onset at 425 nm.

Impact of precursor and crystallization promotor concentration on the film growth

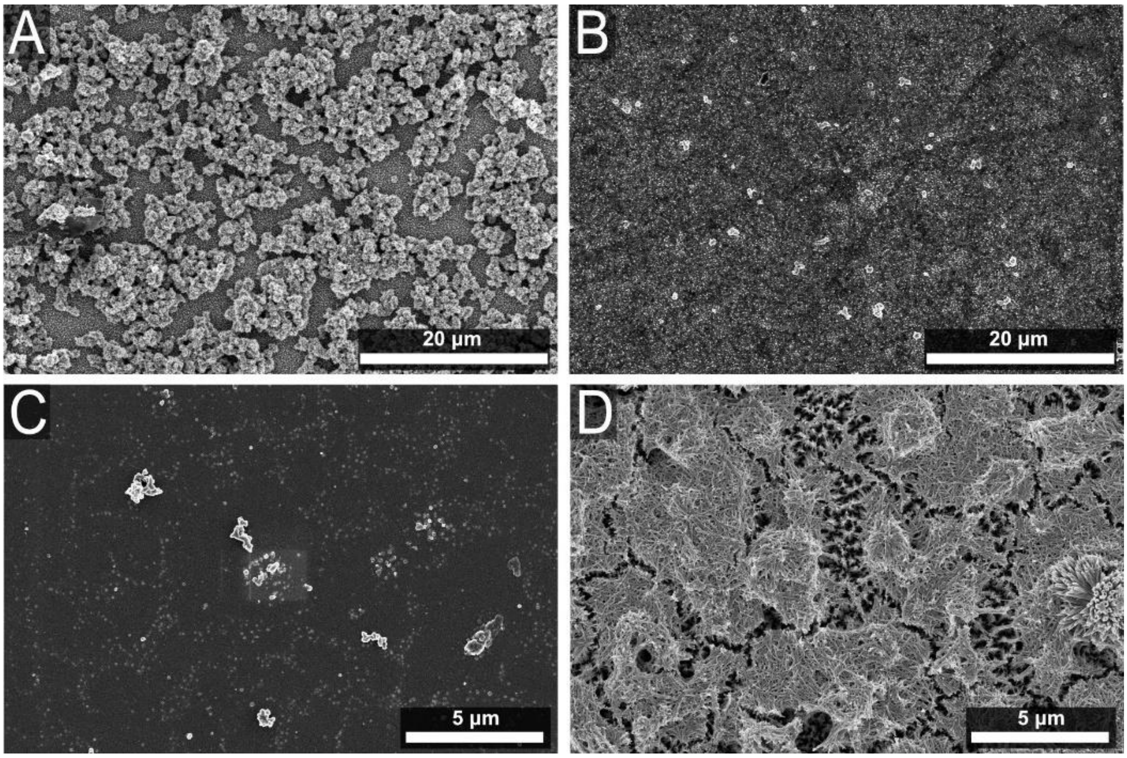

Figure S5.10 Reducing the concentration of the crystallization promotor with respect to the optimal conditions led to uncontrolled formation of bulk material on top of the film **(A)**. A higher concentration of the additive led to the formation of an amorphous phase **(B)**. Reducing the precursor material concentration led to the formation of an amorphous material precipitation on the surface **(C)**, while higher concentrations led to the precipitation of residual powder on the MOF film **(D)**.

6 Anti-fog coating experiments

Macroscopic photograph of the anti-fog coating experiment

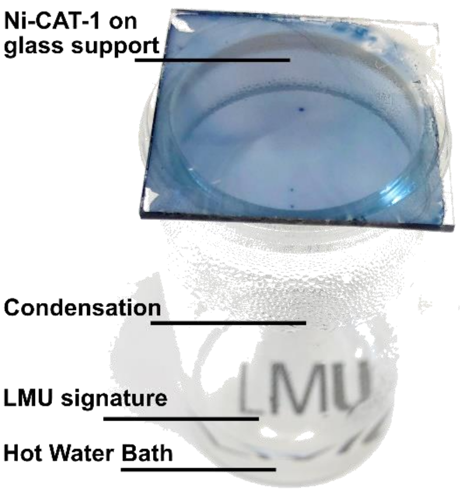

Figure S6.1 In a glass vessel a hot water bath is provided. The hot steam rising from the hot water reservoir is condensing on the upper part of the glass vessel, while the Ni-CAT-1 coated glass support remains free of water droplets. Therefore, vision through the glass support is enabled.

Temperature-dependent anti-fog coating experiments

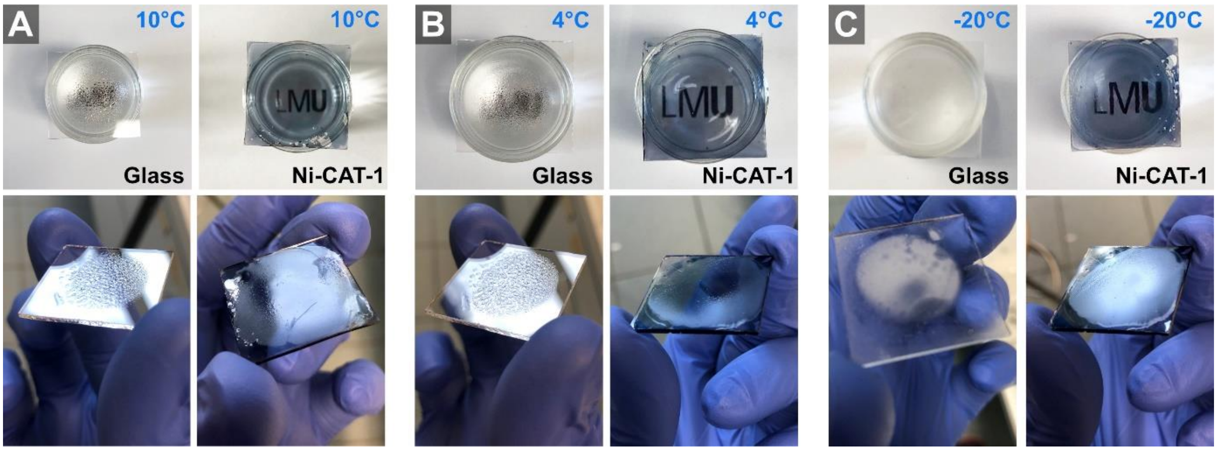

Figure S6.2 Temperature-dependent anti-fog experiment series. Here, glass reference and the Ni-CAT-1 film on glass were cooled down to temperature of 10 °C, 4 °C and -20 °C. After cooling, the substrates were placed on top of the hot water reservoir. It can be seen that in all cases the hot vapor steam is not condensing on the Ni-CAT-1 glass supported samples.

7 Wetting models

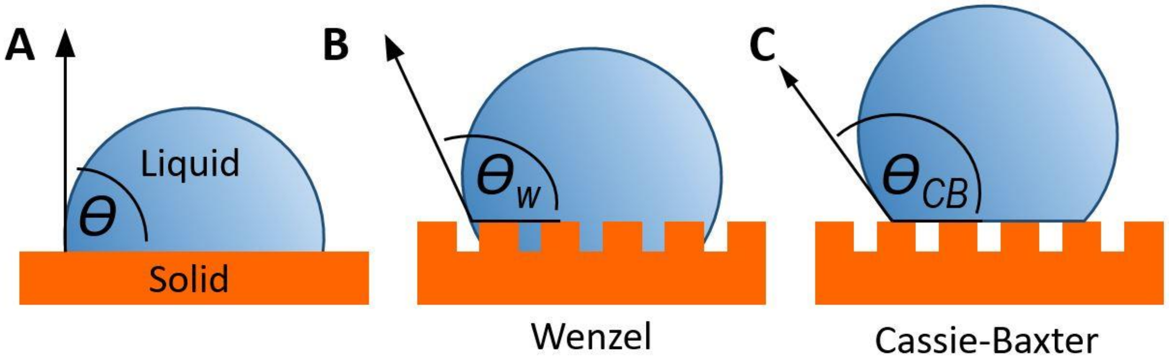

Figure S7.1 A Scheme of possible wetting models for a liquid of a structured solid surface. **A)** Displays the wetting angle Θ of a theoretical flat surface. **B)** The change of the wetting angle to Θ_w according to the Wenzel equation: $\cos\Theta_w = r \cdot \cos\Theta$. In this model, the structuring of the solid enhances the inherent existing wetting properties of the surface. The liquid is homogenously wetting the surface. **C)** a Cassie and Baxter description of the contact angle Θ_{CB} referring to a heterogeneous wetting of the surface by interacting solely with the elevated structured surface.

8 Stability Test

Ni-CAT-1 oriented and nanostructured films on gold substrates

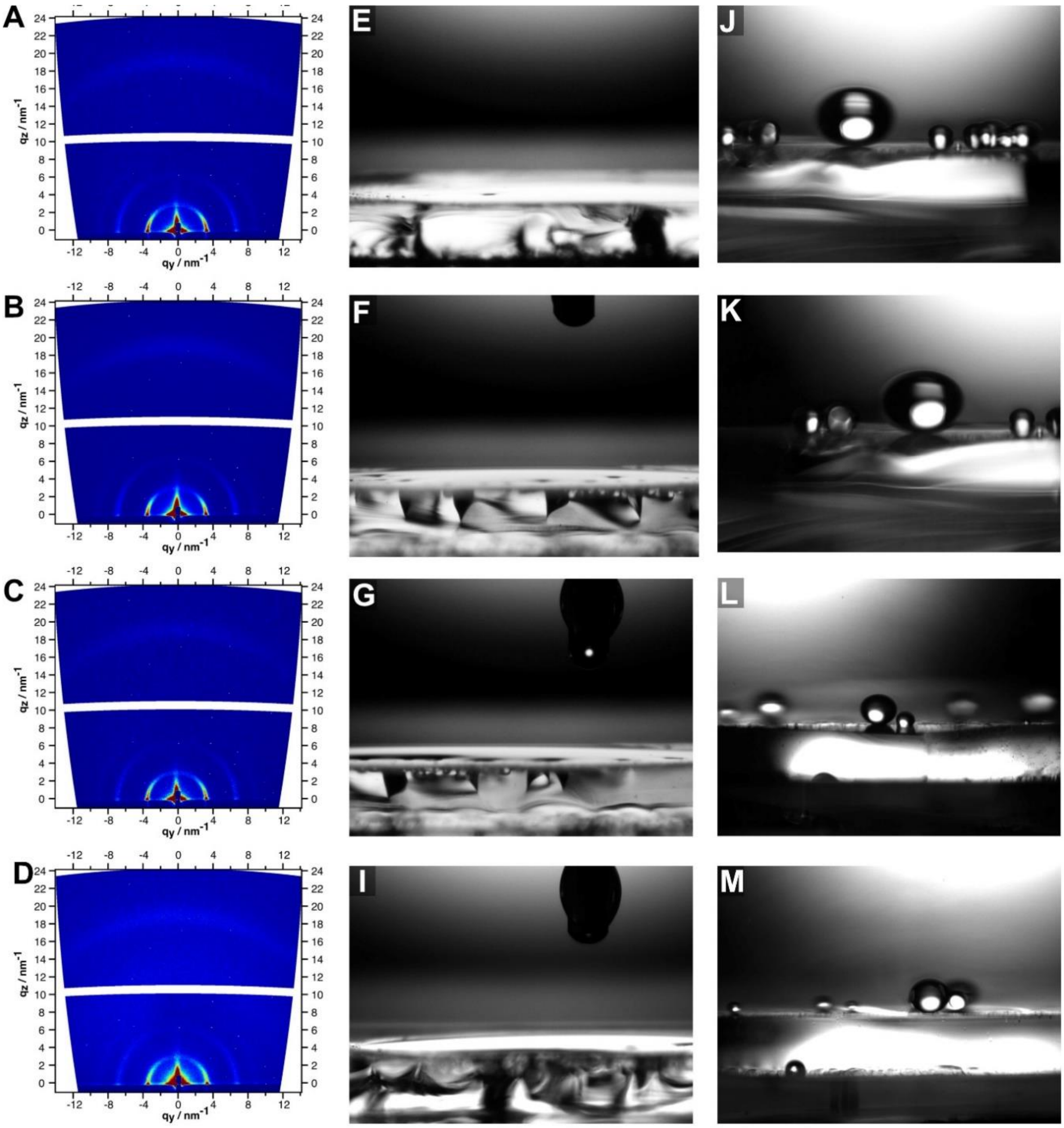

Figure S8.1 A), B), C) and **D)** GIWAXS patterns of nanostructured Ni-CAT-1 thin films on gold substrates after immersion in an acidic (hydrochloric acid) aqua solution (pH 5, 12 h), basic (sodium hydroxide) aqua solution (pH 9, 12 h), exposure to high temperatures (150 °C, 12 h), and mechanical impact (sonication in a water bath, 20 min), respectively. **E), F), G)** and **H)** WCA of Ni-CAT-1 nanostructured thin films after immersion in an acidic (hydrochloric acid) aqua solution (pH 5, 12 h), in basic (sodium hydroxide) aqua solution (pH 9, 12 h), exposure to high temperatures (150 °C, 12 h), and mechanical impact (sonication in a water bath, 20min), respectively. **J), K), L)** and **M)** OCA of Ni-CAT-1 nanostructured thin films after immersion in an acidic (hydrochloric acid) aqua solution (pH 5, 12 h), in basic (sodium hydroxide) aqua solution (pH 9, 12 h), exposure to high temperatures (150 °C, 12 h), and mechanical impact (sonication in a water bath, 20 min), respectively.

Co-CAT-1 oriented and nanostructured films on gold substrates

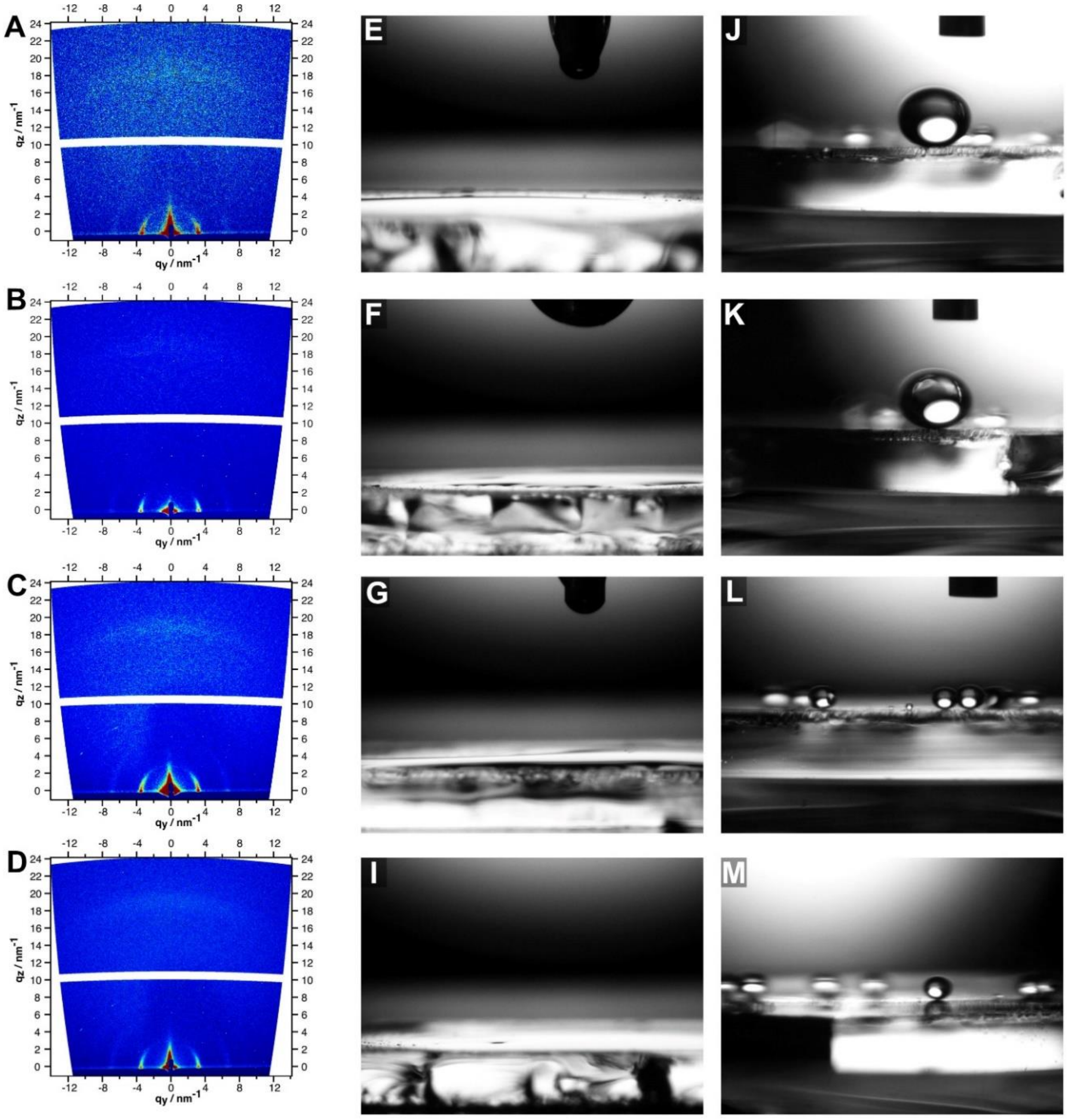

Figure S8.2 A), B), C) and **D)** GIWAXS patterns of nanostructured Co-CAT-1 thin films on gold substrates after immersion in an acidic (hydrochloric acid) aqua solution (pH 5, 12 h), basic (sodium hydroxide) aqua solution (pH 9, 12 h), exposure to high temperatures (150 °C, 12 h), and mechanical impact (sonication in a water bath, 20 min), respectively. **E), F), G)** and **H)** WCA of Co-CAT-1 nanostructured thin films after immersion in an acidic (hydrochloric acid) aqua solution (pH 5, 12 h), basic (sodium hydroxide) aqua solution (pH 9, 12 h), exposure to high temperatures (150 °C, 12 h), and mechanical impact (sonication in a water bath, 20min), respectively. **J), K), L)** and **M)** OCA of Co-CAT-1 nanostructured thin films after immersion in an acidic (hydrochloric acid) aqua solution (pH 5, 12 h), basic (sodium hydroxide) aqua solution (pH 9, 12 h), exposure to high temperatures (150 °C, 12 h), and mechanical impact (sonication in a water bath, 20 min), respectively.

Nanostructured Ni-CAT-1 films on glass substrates

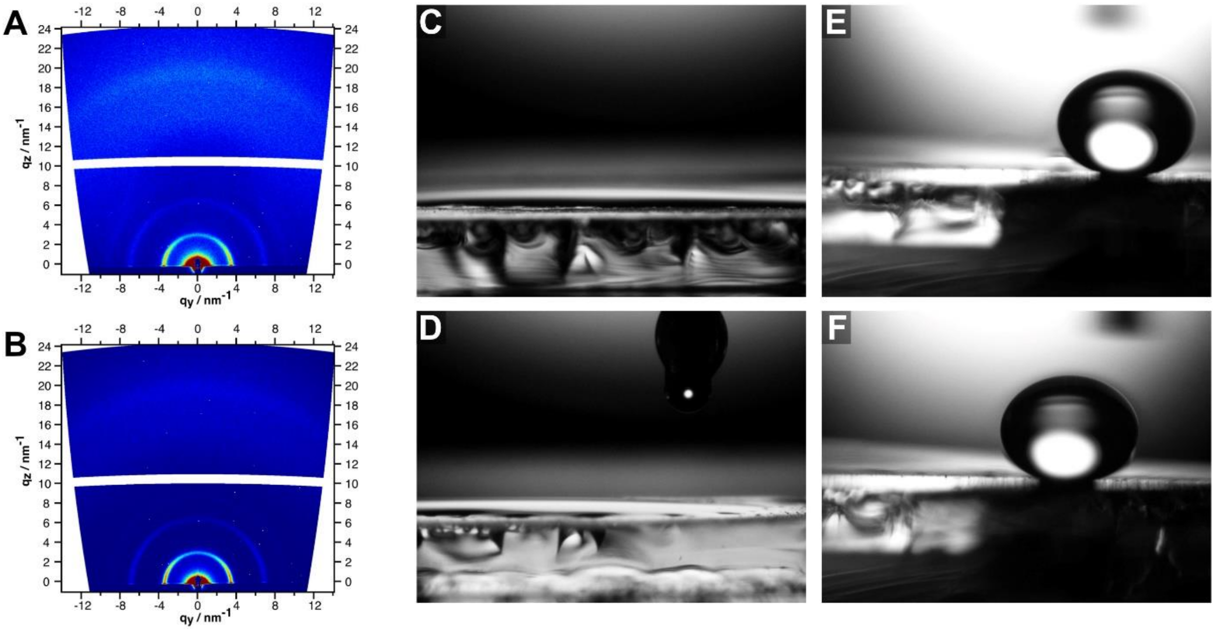

Figure S8.3 A), B) GIWAXS patterns of nanostructured Ni-CAT-1 thin films grown on gold substrates after immersion in an acidic (hydrochloric acid) aqua solution (pH 5, 12 h) and exposure to high temperatures (150°C, 12 h), respectively. **C), D)** WCA of Ni-CAT-1 nanostructured thin films after immersion in an acidic (hydrochloric acid) aqua solution (pH 5, 12 h) and exposure to high temperatures (150 °C, 12 h), respectively. **E)** and **F)** OCA of Ni-CAT-1 nanostructured thin films after immersion in acidic (hydrochloric acid) aqua solution (pH 5, 12 h) and exposure to high temperatures (150°C, 12h), respectively.

References

S1. Mähringer, A.; Jakowetz, A. C.; Rotter, J. M.; Bohn, B. J.; Stolarczyk, J. K.; Feldmann, J.; Bein, T.; Medina, D. D. *ACS Nano* **2019,** *13*, 6711–6719.
